# Red single wavelength emitting up-conversion nanoparticles modulate cellular dynamics and gene expression in T24 bladder cancer cells

**DOI:** 10.1038/s41598-025-24455-0

**Published:** 2025-11-27

**Authors:** Asmaa R. Alsayeh, E. M. Abdelrazek, A. H. Oraby, Ahmed A. Shokeir, Raghda Abo Gabal

**Affiliations:** 1 Department of Physics, Faculty of Science, Mansoura University, Mansoura, 35516 Egypt; 2 Center of Excellence for Genome and Cancer Research, Urology and Nephrology Center, Mansoura University, Mansoura, 35516 Egypt

**Keywords:** Biophysics, Cancer, Drug discovery, Molecular biology, Medical research, Molecular medicine, Urology, Materials science, Nanoscience and technology

## Abstract

Bladder cancer persists in posing a significant global health challenge, highlighting the need for the development of advanced therapeutic strategies. This study investigates the effects of red single-wavelength upconversion nanoparticles (UCNPs), co-doped with Sm^3^⁺ and Nd^3^⁺ in a NaYbF_4_ matrix, on T24 bladder cancer cells. The UCNPs were synthesized using a thermal decomposition method and characterized for their optical, structural, and morphological properties. The nanoparticles exhibited strong red emission under 980 nm near-infrared (NIR) excitation. Cytotoxicity assays revealed concentration-dependent cell death, with enhanced effects under radiation. Wound healing assays demonstrated that UCNPs reduced cellular repair mechanisms, with radiation further enhancing this effect. Gene expression analysis revealed significant modulation of key genes involved in cancer progression, including upregulation of IL-6, and downregulation of anti-apoptotic genes such as BCL2, HIF, and Survivin. Additionally, UCNPs raised the levels of reactive oxygen species (ROS), indicating oxidative stress. These findings highlight the therapeutic potential of UCNPs in bladder cancer treatment.

## Introduction

Bladder cancer is one of the most recurrent malignancies in developed countries^1^. A variety of therapeutic modalities have been implemented in clinical practice for cancer treatment including chemotherapy, surgery, immunotherapy, and molecularly targeted therapies. These strategies exert anticancer effects through mechanisms such as inhibition of cell proliferation, suppression of angiogenesis and metastasis, induction of apoptosis, and reversal of multidrug resistance. In parallel, photobiomodulation (PBM) has been explored as an adjunctive approach to mitigate cancer therapy associated complications. However, its application in oncology remains controversial, as the biological effects of light exposure on cancer cells are still poorly understood^2^. Previous studies examining the effect of multiple wavelengths on cancer biology often attributed cellular outcomes directly to the light source. For instance, blue light (BL) and near-infrared (NIR) irradiation were associated with tumor-promoting effects in malignant melanoma through the upregulation of EGFR, HIF-α, VEGF, and MMP-9^3^. Yet, growing evidence suggests that these outcomes cannot be explained solely by light exposure. Instead, the interaction of light with nanomaterials particularly upconversion nanoparticles (UCNPs) plays a pivotal role in shaping the cellular response^4,5^.

UCNPs have garnered considerable attention due to their unique optical properties, including the ability to convert that allow them to absorb low-energy NIR photons (NIR, 700 nm to 1 mm)and convert them into higher-energy emissions in the visible (VIS, 400 nm to 700 nm)or ultraviolet range (UV, 10 nm to 400 nm)^6–11^. Yb and Nd co-doped upconversion nanoparticles (UCNPs) have gained significant attention for applications in bioimaging, sensing, energy modulation, and anticounterfeiting. Their unique optical feature lies in the ability to absorb two or more near-infrared (NIR) photons sequentially and re-emit them as higher-energy light in the ultraviolet, visible, or NIR regions. This property makes them particularly valuable for biological studies, as it allows imaging with deeper tissue penetration and minimal background interference^12^, making UCNPs suitable for biomedical applications^13–26^. Beyond their imaging utility, UCNPs can serve as mediators of light-induced biological effects by channeling energy into specific molecular pathways. Achieving efficient upconversion with emission restricted to a single red wavelength involves addressing several critical factors (such as, dopant ion selection and concentration^27^, host lattice and crystal phase^28^, surface modification and core–shell design^29^, and particle size and morphology^30^). For example, β-NaYF_4_:Yb^3+^/Ho^3+^@β-NaYbF_4_:Er^3+^ core/shell nanorods were employed to achieve red up-conversion emission under 980 nm laser excitation with (0.2 W mm⁻^2^). Emission bands centered at 519, 538, and 654 nm were observed, corresponding to the ^2^H₁₁_/_₂ → ^4^I₁₅_/_₂, ^4^S₃_/_₂ → ^4^I₁₅_/_₂, and ^4^F₉_/_₂ → ^4^I₁₅_/_₂ transitions of Er^3^⁺, respectively, with a notably intense red emission^31^. However, the complexity of the seed-mediated synthesis approach poses challenges, including limited control over nanoparticle size. Cubic-to-hexagonal phase transition process using oleylamine, acting as a surface capping ligand, regulates the growth of NaYbF_4_ nanoparticles by facilitating the transformation of cubic-phase intermediates into hexagonal-phase products. Although this method yields small, tunable-sized NaYbF_4_ nanoparticles, their red upconversion emission remains weak^32–35^. The toxicity of UCNPs has been studied concerning in vitro cytotoxic activity and long-term in vivo toxicity^36^. Despite these advancements, the effects of UCNPs on cancer cells after infrared red exposure remain underexplored, highlighting the need for further research to fully understand their potential in cancer therapy^37^. Subtle changes in the type or amount of lanthanide ions can significantly influence the overall upconversion performance. These changes affect not only emission intensity and colour but also energy transfer dynamics and the efficiency of the excited-state processes. For example, previous studies have shown that even slight variations in Er^3^⁺ content within ZrO₂ matrices can lead to marked shifts in luminescent behaviour due to phase transitions and local structural changes in the crystal lattice^38^. Obayashi has Irradiated pancreatic cancer cells using a 915‑nm laser significantly that induced caspase‑3 activation and apoptosis^39^.

While much progress has been made in tailoring UCNPs for photonic and imaging purposes, less is known about how their optical emissions influence cancer cell biology. Red-emitting UCNPs, in particular Red single-wavelength UCNPs, have shown potential in biomedical applications owing to their biocompatibility, tunable emission properties, and ability to induce photodynamic or photothermal effects. Phototherapy efficiently generates heat photothermal therapy (PTT) or reactive oxygen species (ROS) such as singlet oxygen photodynamic therapy (PDT). These nanoparticles can generate localized heating, which disrupts cancer cell protein structures and DNA, inducing apoptosis and enhancing the cells’ sensitivity to radiation and chemotherapy^40,41^. Hyperthermia upregulates heat shock proteins (HSPs) on cancer cells, acting as danger signals that promote antigen presentation and enhance immune recognition^42^. Elevated temperatures activate macrophages, promoting the release of pro-inflammatory cytokines like IL-1β, IL-6, and TNF-α, which recruit and activate other immune cells to strengthen the immune response against tumors^43,44^. Despite these promising effects, previous studies have not thoroughly investigated the specific molecular responses of cancer cells to red light alone or the gene expression changes and potential synergistic effects with other therapies. Despite these promising mechanisms, the specific molecular and genetic responses of cancer cells to red single-wavelength UCNPs remain insufficiently explored^45,46^.

In this study, we investigate a novel red-emitting UCNP composition and assess its biological effects on T24 bladder cancer cells. Additionally, we assess the impact on cell migration and repair mechanisms through wound healing and the influence on reactive oxygen species (ROS) generation. Additionally, the study explores the nanoparticles’ (NPs) impact on the expression of key genes involved in cancer progression and resistance, including interleukin-6 (IL-6), B-cell lymphoma 2 (BCL2), sex-determining region Y-box 2 (SOX2), hypoxia-inducible factor-alpha (HIF-α), Survivin, Caspase 3, transient receptor potential cation channel subfamily V member 4 (TRPV4), Multidrug resistance protein 1 (MDR1), and ATP-binding cassette sub-family C member 1 (ABCC1). By focusing on the nanomaterial–mediated biological effects rather than light alone, this work aims to provide a comprehensive understanding of how red single-wavelength UCNPs modulate cancer cell responses. Ultimately, our findings may contribute to the development of nanomaterial based therapeutic strategies for bladder cancer, with potential applications in precision oncology.

## Materials and techniques

### Materials and chemicals

All purchased materials were used as supplied, without additional purification. Ytterbium(III) nitrate hexahydrate (Yb(NO₃)₃·xH₂O, 99.9%), Samarium(III) nitrate hexahydrate (Sm(NO₃)₃·6H₂O, 99.9%), and Neodymium(III) nitrate hexahydrate (Nd(NO₃)₃·6H₂O, 99.9%) were obtained from Alfa Aesar, Germany. Sodium hydroxide (NaOH), sodium fluoride (NaF), ethanol (C₂H₆O), and oleic acid (OA) were obtained from LANXESS, Germany. 3-(4,5-Dimethyl-2-thiazolyl)-2,5-diphenyl-2H-tetrazolium bromide (MTT) was obtained from SERVA.

### Methods and preparation of β-NaYbF_4_:Sm_y_,Nd_z_

UCNPs were prepared using thermal decomposition method, which is an advanced top-down technique that builds upon the traditional solvothermal approach^47–49^. The main process begins with the preparation of the organometallic complex. This complex is subsequently dissolved in an organic solvent, which is then mixed with a stable surfactant. Afterward, a brief high-temperature thermal decomposition is performed under controlled atmospheric conditions. Finally, the target product is prepared. To prepare highly fluorescent NaYb_x_F_4_:Sm_y_,Nd_z_ UCNPs, two samples were synthesized: one with X = 75, Y = 20, and Z = 6 mol%, and another with X = 80, Y = 15, and Z = 6 mol%. First, a mixed solution was prepared by combining 3 mL of deionized water, 10 mL of ethanol, and 0.6 g of NaOH. Next, 10 mL of oleic acid was added to this mixture while stirring vigorously. The stirring was maintained for 20 min, resulting in a solution referred to as Solution A. 500 mg of Yb(NO_3_)_3_⋅xH_2_O, 130 mg of Sm(NO_3_)_3_⋅6H_2_O, 30 mg of Nd(NO_3_)_3_⋅6H_2_O were dissolved in 4 mL of deionized water, creating Solution B. Solution B was then gradually added to Solution A while stirring vigorously. After 10 min, 2.0 mL of 2M NaF solution was added dropwise to the mixture. This resulted in a milky colloidal solution. The solution was transferred to a 50 mL autoclave flask, heated to 180 °C at a rate of 3 °C/min, and held at 200 °C for 2 h. The synthesized UCNPs were then collected and rinsed with ethanol followed by ultrapure water. The resulting products were obtained by vacuum drying at 70 °C for 12 h.

### Instrumentation and methods

#### Ultraviolet–visible spectroscopy (UV–Vis)

UV–Vis absorption was assessed using Pg instruments, T80+, UV/Vis spectrometer from China that spanning wavelengths from 200 to 900 nm to investigate specimen structure and optical characteristics. This encompassed photon spectroscopy across ultraviolet, visible, near-ultraviolet and near-infrared ranges, in which molecules experience electronic transitions. Both bonding and non-bonding electrons have the ability to absorb visible light or UV energy, prompting excitation to higher antibonding molecular orbitals^50^.

Tauc Law (Eq. 1) was used to estimate the optical band gap (energy gap) of the UCNPs from their UV–Vis absorption spectra it is particularly useful in analyzing the optical properties of materials and understanding their electronic band structure

1 $$\left( {\upalpha {\text{h}}\upnu } \right)^{{\text{n}}} = {\text{ K}}({\text{h}}\upnu - {\text{Eg}})$$

where α represents the absorption coefficient. **hν** denotes the photon energy (with h being Planck’s constant and ν being the frequency of light). **Eg** is the optical band gap (energy gap) of the material. **K** is a constant. **n** is an exponent that varies based on the type of electronic transition (usually 1/2 for direct transitions, 2 for indirect transitions, 2/3 for forbidden direct transitions, 3 for forbidden indirect transitions)^51,52^.

In the Tauc plot method, energy “**hν**” is plotted on the x-axis against (**αhν**)^n^ on the y-axis. By extrapolating the linear portion of the curve, a line is drawn to intersect the x-axis allows for the determination of the material’s optical band gap. The direct bandgap nature of the UCNPs, as indicated by the linear part in the analysis, suggests efficient electronic transitions without the need for phonon assistance, which is advantageous for optoelectronic applications^53^.

#### X-ray photoelectron spectroscopy (XPS)

X-ray photoelectron spectroscopy (XPS) of UCNPs was collected on K-ALPHA (Themo Fisher Scientific, USA) with monochromatic X-ray Al K-alpha radiation (−10 to 1350 e.v) spot size 400 micro m, at pressure (10-9) mbar with full spectrum pass energy 200 e.v and at narrow spectrum 50 e.v^54^.

#### X-ray diffraction (XRD)

X-ray diffraction (XRD) analysis of UCNPs was performed utilizing a Shimadzu XRD-6000 diffractometer from Japan. The source utilized CuKα radiation with a wavelength (λ) of 0.15405 nm and a power of 2 KW. These measurements aimed to ascertain the crystallinity, structural imperfections, texture of the samples, and crystallite size. X-ray diffraction (XRD) patterns were obtained within the 2θ range of 4° to 100°, with a scanning rate of 0.02° per minute. Using smooth double-sided adhesive tape, the dried powder samples were affixed to a sample holder. The nature of the prepared UCNPs was determined through XRD analysis. The crystallite size (D) of a particle was determined using the Scherrer equation, as described in (Eq. 2)^55^.

2 $${D}_{hkl}=\frac{K\lambda }{\beta cos \theta }$$

In this equation, λ denotes the X-ray wavelength, measured at 0.154 nm. D represents the mean diameter of the crystals. K represents the structural factor, with a designated value of 0.89. β denotes the whole width at half maximum of the significant peaks. Where, θ is the Bragg angle, expressed in degrees. The Scherrer equation was employed to ascertain the crystallite size and average crystallite size from the XRD data^56–58^ . The interplanar spacing (d_hkl_) was determined using Bragg’s law as described in (Eq. 3).

3 $${d}_{hkl}=\frac{\lambda }{2 sin \theta }$$

where (d_hkl_) is the interplanar spacing, λ is the X-ray wavelength, and θ is the diffraction angle corresponding to the peak position^55^. The interplanar spacing (d) was calculated using Bragg’s equation for hexagonal systems as described in (Eq. 4)

4 $$\frac{1}{{d}^{2}}= \frac{4}{3}\cdot \frac{{h}^{2}+ hk + {k}^{2}}{{a}^{2}}+\frac{{l}^{2}}{{c}^{2}}$$

where h, k, l are the Miller indices of the reflecting planes, a and c are the lattice parameters of the hexagonal unit cell^59^. Lattice strain results from crystal defects. Since nanoparticles alleviate strain by expanding the lattice, it serves as a quantitative indicator of lattice imperfections and dislocations. In XRD analysis, broadening of the peak occurs due to strain induced by lattice distortion. The Williamson Hall (W–H) method is commonly employed to calculate lattice strain, as described in (Eq. 5)^60^.

5 $$\beta_{hkl} \cos \theta = \frac{k\lambda }{D} + 4\varepsilon \sin \theta$$

Here, ε represents the lattice strain of particles, measured in radians. By plotting 4sinθ on the X-axis and βhkl cosθ on the Y-axis, a linear relationship is established. The slope of the fitted line corresponds to ε, while the intercept represents D.

In Selected Area Electron Diffraction (SAED), the electron beam interacts with the atomic arrangement of the sample, producing characteristic diffraction patterns. The radii (R) of the resulting diffraction rings were measured and used to determine the interplanar spacing (d-spacing) associated with the corresponding crystal planes^55,61^.

#### Fourier transform infrared spectroscopy (FTIR)

Spectra from FTIR were examined utilizing a FTIR spectrophotometer (PerkinElmer 99075, from Germany), which employed the fundamental KBr pellet method within the spectrum range of 4000 to 450 cm^−1^, achieving a resolution accuracy of 4 cm^−1^. The samples were pulverized, mixed with KBr, and subsequently formed into pellets. Fourier transform infrared (FT-IR) spectroscopy was employed to ascertain the functional groups of the active constituents involved in the synthesis of NaYbF_4_:Nd^+3^,Sm^+3^^62^.

#### Transmission electron microscopy (TEM)

Transmission electron microscopy studies were conducted with a JEM-2100 system from JEOL Ltd. company Japan, integrated with a CCD camera, and running at an acceleration voltage of 200 kV. Samples were diluted before imaging. A copper grid was positioned on a wax plate, and once the grid dried, a drop of the diluted nanocolloidal suspension was applied to it. The size distribution of nanoparticles was examined using ImageJ (64-bit) program. Selected Area Electron Diffraction (SAED) was performed to assess the lattice characteristics, crystallinity of the samples, and their orientation. SAED involves the interaction of electron beams with the sample’s atoms, leading to the formation of diffraction patterns. The radii of the rings in these diffraction patterns were measured, which correspond to the d-spacing of the crystal planes^61^. The diameters of the SAED rings were measured using ImageJ software, and the corresponding d-spacings were calculated using Bragg’s law (Eq. 3), The calculated d-spacing values were then compared with standard reference data to assign the corresponding Miller indices (hkl) to each ring^55^.

#### Energy-dispersive x-ray (EDX) microanalysis

EDX was employed to examine the composition of the sorbent, with samples positioned on aluminum foil within a sample container. Analysis was performed utilizing a Hitachi SEM 3200 electron microscope integrated with an EDS detector. Additionally, the UCNPs structure was characterized using an EDX spectrum via an x-ray micro-analyzer (silicon drift detector with 129 eV resolution energy) attached to a JEOL JSM IT-100 scanning electron microscope (SEM). This confirmed the presence of lanthanides and detected other elemental compositions^63^.

#### Surface roughness and waviness calculation

The surface roughness of UCNPs was quantitatively analyzed using ImageJ software, considering several key roughness parameters to evaluate surface texture. These parameters provide detailed insights into how surface characteristics deviate from the mean line, allowing for a comprehensive assessment of the material’s topography.

Key roughness parameters include Arithmetical Mean Deviation (Ra), also known as average roughness, which represents the mean value of the amplitudes relative to a reference line. The Ra values were determined using the following (Eq. 6)^64^:

6 $$\text{Ra }=\frac{1}{L}\underset{0}{\overset{L}{\int }}f\left(x\right)dx$$

where L is the evaluation length and f(x) represents the roughness profile. The Sampling Length (l), also called the Cutoff Length, defines the nominal wavelength threshold that distinguishes roughness from waviness, while the Evaluation Length (L) refers to the total span over which surface roughness parameters are measured. Root Mean Square Deviation (Rq), also called root mean square roughness, is the square root of the squared amplitudes relative to the midline. Kurtosis (Rku) measures the tip geometry of peaks and valleys, indicating the profile’s peakedness and helping to analyze the degree of contact between two surfaces. Skewness (Rsk) evaluates the asymmetry of the profile about the mean line. The Lowest Valley Depth (Rv or Zv) represents the depth of the largest valley, determined by minimum measurements. The Maximum Profile Peak Height (Rp) quantifies the distance from the peak of the profile to the average line within the evaluation length. Finally, the Total Height of the Profile (Rt) represents the vertical distance between the highest and lowest points within the evaluation length^65^. Waviness parameters describe the broader, more periodic surface features that lie between the fine texture of roughness and the overall shape of a surface. A larger Rt indicates a more undulated surface. Other parameters such as mean height of the profile elements (Rc), frequency of profile occurrences (FPO), and surface area (SA) also help in understanding the frequency and distribution of these waviness features across the surface.

#### Zeta potential

The zeta potential of the UCNPs suspension was measured at 25 °C with the Malvern Zetasizer Nano-ZS 90 (USA), a zeta potential analyzer and particle sizing instrument. A scattering angle of 90° was employed. Prior to measurement, the UCNP suspensions were diluted with deionized water. In zeta potential analysis, charged colloidal dispersions were placed into a zeta cell, a glass cuvette with rounded ends^66^.

Particle size measurements were performed with a dynamic light scattering apparatus (DLS) (Zeta Potential Analyzer/Particle Sizing Systems PSS.NICOMP 380 ZLS, USA), with assessments completed at a scattering angle of 90°^66^.

#### Photoluminescence spectroscopy (PL)

The luminescence properties were assessed using a fluorospectrophotometer (F-2500, HITASCHI) and two-photon laser confocal microscopy (690–1040 nm) (CLSM, Carl Zeiss LSM710). The luminescence properties of the nanocomposite were analyzed by studying its excitation and emission spectra^67^.

#### Hemolysis assay

Blood samples from volunteers were diluted with 10 mL of Phosphate buffer saline (PBS), PH = 7.4, and the red blood cells (RBCs) were isolated from the serum using centrifugation at 1200 rpm for 10 min. The red blood cells were washed at least four times and subsequently resuspended in 10 mL of PBS. Subsequently, 200 µL of the diluted RBCs solution was added to 800 µL of PBS (to serve as a negative control), 0.1% wt Sodium Dodecyl Sulfate (SDS) (to serve as a positive control), and different concentrations of UCNPs1 and UCNPs2 (400, 200, 100, 50, and 25 µg/mL). The mixtures were incubated at 37 °C for 4 h, subsequently the mixture was centrifuged at 12,000 rpm for 5 min. The supernatants from all samples were collected and the absorbance was measured at 541 nm using a UV–VIS spectrophotometer (NanoDrop 2000c). The hemolysis percentage in the red blood cells was subsequently determined using the hemolysis ratio calculation formula (Eq. 7)^68^.

7 $${\text{hemolysis ratio }}\left( \% \right) \, = \, \left[ {\left( {{\text{A}}_{{{\text{sample}}}} - {\text{ A}}_{{\text{negative control}}} } \right)/\left( {{\text{A}}_{{\text{Positive control}}} - {\text{ A}}_{{\text{negative control}}} } \right)} \right] \, \times { 1}00\%$$

To assess cellular integrity during the hemolysis assay, trypan blue staining was employed to distinguish between intact and lysed red blood cells. A 1:1 volume ratio was used by mixing 3 µL of trypan blue with 3 µL of each red blood cell sample (including all treated groups, as well as negative and positive controls). The mixture was gently placed onto a clean glass microscope slide. After allowing the dye to incubate for 1 min at room temperature, imaging was performed immediately to minimize potential staining artifacts. All samples were examined under a light microscope using a 20× objective lens, corresponding to a scale of 200 µm. This approach enabled the visualization of non-lysed cells with intact nuclei, which excluded the dye, providing a clear contrast between viable and damaged cells across all experimental groups.

### Cellular experiments

#### In vitro cytotoxicity and cell viability assays (MTT assay)

A stock solution was prepared using 5 mg of prepared NaYb_x_F_4_:Sm_y_,Nd_z_ dissolved in 1 ml consists of 1.5% DMSO and 98.5% DMEM and kept at − 20 °C. A human bladder cancer (T24) cell line was obtained from NAWAH, Egypt. The T24 cell line was grown in a tissue culture flask containing DMEM high glucose Biowest, Canada. The culture medium comprised 10% fetal bovine serum (FBS) from Gibco, California and 100 IU/mL of penicillin and 100 μg/mL streptomycin from Gibco BRL, New York. The cells were incubated at 37 °C in a 5% CO_2_ environment with high humidity. The medium was replaced every three days until the cells reached 70 to 80% confluency. Once this confluency was achieved, the cells were trypsinized with 0.25% trypsin–EDTA and subsequently counted. The cells underwent trypsinization using trypsin/EDTA and were subsequently resuspended in an equivalent volume of fresh medium. A 10 µL aliquot of the cell suspension was then placed in a 1.5 mL Eppendorf tube. Subsequently, 10 µL of Trypan blue at a concentration of 0.4% was added to the tube and gently mixed. The hemocytometer underwent thorough cleaning, and the coverslip was moistened with water. Following that, 10 µl of the cell suspension was placed into each chamber, and the samples were analyzed using a microscope with 10× magnification. The concentration of viable cells was determined using (Eq. 8):

8 $$\text{Average living cell count}=\frac{\text{total number of living cells }}{\text{total number of squares }\times \text{ dilution factor }\times {10}^{4}}$$

Cells were plated in 96-well plates at a density of 5 × 10^4^ cells/mL, with 100 µL dispensed into each well. Following overnight incubation at 37 °C in a 5% CO_2_ atmosphere, the cells were treated with varying concentrations of UCNPs1 and UCNPs2 (500, 250, 125, 62.50, 31.25, 15.62, 7.81, and 3.90 µg/ml), alongside a negative control containing 1.5% (v/v) DMSO, and subsequently incubated for 24 h. The cells were subsequently treated differently based on the presence or absence of near-infrared radiation. In the radiation group, following an initial 24-h incubation, the cells were exposed to near-infrared light at 980 nm and then incubated for an additional 48 h. Subsequently, 100 µL of 3-(4,5-dimethylthiazol-2-yl)-2,5-diphenyl-2H-tetrazolium bromide (MTT) solution prepared at a concentration of 5 mg/mL in PBS, was then added to each well for both groups, and the mixture was incubated for 4 h. The living cells transformed MTT into formazan crystals, subsequently dissolved by the addition of 100 µL of DMSO and incubation for 15 min at 37 °C in a 5% CO_2_ environment. Absorbance was measured at a wavelength range of λ = 570–630 nm utilizing an Infinite F50 plate reader. Each experiment was performed in triplicate, and cell viability was evaluated using (Eq. 9). IC_50_ values were calculated utilizing GraphPad Prism 8 program.

9 $$\text{Cell viability }=\frac{(\text{ optical density of the sample }(\text{ODSample}) -\text{ optical density of the blank }(\text{ODblank}))}{(\text{ optical density of the control }(\text{ODcontrol}) -\text{ optical density of the blank }(\text{ODblank}))}*100\text{\%}$$

#### Wound healing (scratch) assay

Bladder cancer cells (T24) were grown in six-well plates until each well reached 90% confluence. A linear wound was created in the cell layer using a 200 µl pipette tip. Detached cells were eliminated by rinsing with PBS. The plates were then incubated in DMEM medium lacking fetal bovine serum for 24 h. Following this, the IC50 concentrations of UCNPs1, UCNPs2, radiated UCNPs1, and radiated UCNPs2 were introduced into their respective wells. At 24 h representative scratch zones for every cell line have been captured by the lecia microscope (Wetzlar, Germany), a 10× objective. Each experiment was performed in triplicate for each concentration UCNPs1, UCNPs2 and untreated (Control). To assess the results, micrograph was analysed using Wound Healing Tool plugin in ImageJ software. For T24 cells, the migration process happened through the scratched area. This was the area counted within a 200 × 200 µm frame. Wound Healing % or Wound Contraction % is a parameter used to quantify the rate of wound healing over time. It measures the reduction in wound size as the tissue regenerates and contracts. The formula for calculating Wound Closure % is (Eq. 10):

10 $$\text{Wound Closure \%}=\frac{\text{Initial Wound Area }\left(\text{T}0\right)-\text{Final Wound Area }\left(\text{T}24,\text{ T}48\text{ R}\right)}{\text{Initial Wound Area }\left(\text{T}0\right)}\times 100$$

where the Initial Wound Area is the area of the wound at the beginning of the experiment. And the Current Wound Area is the area of the wound at any given time during the healing process. This calculation helps in assessing the effectiveness of different treatments or conditions in promoting wound healing. The greater the percentage, the more healing has occurred. Negative values in Wound Closure % indicate that the wound has increased in size instead of healing. While positive value shows that the wound area has decreased compared to its initial size, reflecting tissue regeneration and wound closure.

#### Bladder (T24) matrix proteins

##### Analytical tools for studying protein–protein (PPI) and chemico-protein interactions using computational methods

STRING integrates data from several key sources, including experimental interaction data, computationally curated complexes and pathways from existing databases, automated text mining of scientific literature, genomic context predictions, and cross-species evidence. These resources are available through the STRING database. Interaction scores in STRING range between 0 and 1, with a score of 1 representing the highest level of confidence. Moreover, the protein–protein interaction (PPI) networks created by STRING can be visualized using the Cytoscape platform, which aids in achieving a detailed understanding of the interaction landscape^69^.

#### Gene expression assay

T24 bladder cancer cells were seeded in 6-well plates at a density of 2 × 10^5^ cells per well for PCR analysis. The experimental design included six treatment groups: (a) untreated control, (b) cells treated with UCNPs1, (c) cells treated with UCNPs2 all incubated for 24 h; (d) Radiation only (IR), (e) UCNPs1 combined with IR, and (f) UCNPs2 combined with IR. For groups (e) and (f), UCNPs were administered 24 h prior to irradiation and maintained in culture for an additional 24 h post-irradiation. PCR analysis was performed at the IC₅₀ concentrations of UCNPs1, UCNPs2, radiated UCNPs1, and radiated UCNPs2 as established by the preceding MTT viability test. Each condition was conducted in triplicate. Following the treatments, the cells were harvested, and total RNA was extracted via TransZol Up. Adherent cells were lysed using TransZol Up after washing with PBS. The lysate was transferred to a microcentrifuge tube, mixed thoroughly, and incubated for five minutes. RNA was extracted by adding chloroform, followed by centrifugation at 10,000×*g* for 15 min (2–8 °C). The RNA-containing phase was collected, mixed with ethanol, and purified using a spin column. After washing and centrifugation, RNA was eluted in RNase-free water (25 µl), and its purity and concentration were measured using a NanoDrop 200c. Samples were stored at − 8 °C.

For cDNA synthesis, the EasyScript^®^ First-Strand cDNA Synthesis SuperMix kit was used. A reaction mixture (20 µl) was prepared with total RNA (0.1 ng–5 µg), 2× ES Reaction Mix, Anchored Oligo(dT)18 Primer, EasyScript^®^ RT/RI Enzyme Mix, and RNase-free water. To enhance efficiency, an optional pre-incubation at 65 °C for 5 min was followed by cooling on ice. The reaction was then carried out at 42 °C for 15 min and was terminated by heating to 85 °C for 5 s qPCR was conducted in a 20 µl reaction containing cDNA, 0.4 µl of each primer (10 µM), 10 µl of 2 × TransStart^®^ Top/Tip Green qPCR SuperMix, nuclease-free water, and an optional Passive Reference Dye. The cycling conditions included initial denaturation at 94°C for 30 s, followed by 40–45 cycles of 94 °C for 5 s, 50–60 °C for 15 s, and 72 °C for 10 s, with a dissociation stage. In the two-step qPCR protocol, annealing and extension were combined at 60 °C for 30 s. RT-PCR was performed in a 50 µl reaction with cDNA, 1 µl of each primer (10 µM), 25 µl of 2 × TransTaq® HiFi PCR SuperMix II, and nuclease-free water. The cycling conditions consisted of an initial denaturation at 94 °C for 2–5 min, followed by 35–40 cycles of 94 °C for 30 s, 50–60 °C for 30 s, and 72 °C for 1–2 kb/min, with a final extension at 72 °C for 5–10 min.

##### Conventional polymerase chain reaction (cPCR)

Conventional PCR (cPCR) is a widely used technique to amplify specific DNA sequences, enabling the study of gene expression. Several genes of interest in various biological contexts include GAPDH (Glyceraldehyde-3-Phosphate Dehydrogenase), a housekeeping gene with stable expression in most cells, commonly used as a reference gene in gene expression studies. HIF-α (Hypoxia-Inducible Factor 1-α) regulates the cellular response to hypoxia (low oxygen), playing a critical role in angiogenesis and tumor growth in hypoxic environments. SOX2 (SRY-Box Transcription Factor 2) indicate the presence of stem cells or cellular differentiation processes, important in developmental biology and regenerative medicine. Survivin (BIRC5), a member of the Inhibitor of Apoptosis (IAP) family, is crucial for preventing programmed cell death and supporting cell division. It is frequently expressed in cancers and serves as a marker for diagnosing and monitoring tumor progression. BCL2 (B-Cell Lymphoma 2) is an anti-apoptotic gene associated with increased cell survival, often studied in cancer research where it may be expressed in treatment-resistant cancer cells. IL6 (Interleukin 6) A gene associated with inflammation and immune response. IL6 expression can be an indicator of inflammation or immune-related diseases, Transient receptor potential vanilloid 4 (TRPV4).

A 1% agarose gel was prepared by dissolving 0.5 g of agarose in 50 ml of AE buffer, heating for 1.5 min, and adding 2.5 µl of ethidium bromide before pouring it into a gel tray to solidify. Gel electrophoresis is essential for DNA analysis, separating fragments by size under an electric field. Smaller fragments migrate further, and after staining, DNA bands become visible under UV light. This process is essential for applications such as gene expression studies, genetic fingerprinting, and mutation detection.

##### Real-time quantitative PCR (RT-PCR)

The PerfectStart^®^ Green qPCR SuperMix kit was used for conducting the RT-PCR reactions. The reaction mixture was prepared containing 2.5 µl of the cDNA, 12.5 µl of green SuperMix, 1.25 µl of forward primer, 1.25 µl of reverse primer, 7.5 µl of nuclease-free water where the total reaction will be 25 µl. The Rotor-Gene Q MDX real-time PCR system was used to measure the relative expression levels of the genes GAPDH, HIF-α, SOX2, SURVIVIN, BCL2, IL6, and TRPV4. PCR reactions for each gene were conducted in triplicate, and the specificity of the PCR products was verified using melt curve analysis. The housekeeping gene glyceraldehyde 3-phosphate dehydrogenase (GAPDH) was used as an internal control for normalizing the relative expression levels of the target genes. The primer sequences and their corresponding annealing temperatures used in the PCR are listed in Table 1. The expression analysis can be Estimated using (Eq. 11)^70^. *All gene expression values are presented as relative fold changes, calculated by the 2*^*–ΔΔCt*^ method, and normalized to GAPDH as the internal control, and untreated controls were set to 1.0.

**Table 1:** Primer sequences used for gene expression analysis.

Gene	Sequence
GAPDH Glyceraldehyde-3-phosphate dehydrogenase	F: TGCTGGCGCTGAGTACGTCG
	R: TGACCTTGGCCAGGGGTGCT
IL-6 Interleukin-6	F: AGACAGCCACTCACCTCTTCAG
	R: TTCTGCCAGTGCCTCTTTGCTG
SURVIVIN Baculoviral IAP repeat-containing 5	F: ACCGCATCTCTACATTCAAG
	R: CAAGTCTGGCTCGTTCTC
SOX2 SRY-box transcription factor 2	F: GAGGAAGAGGTAACCACA
	R: GCAGTACAACTCCTGAC
HIF-α Hypoxia-inducible factor-alpha	F: GTGGATTACCACAGCTGA
	R: GCTCAGTTAACTTGATCCA
Caspase 3 Caspase 3	F: GGAAGCGAATCAATGGACTCTGG
	R: GCATCGACATCTGTACCAGACC
BCL2 B-cell lymphoma 2	F: GTACTTAAAAAATACAACATCACAG
	R: CTTGATTCTGGTGTTTCCC
TRPV4 Transient receptor potential cation channel subfamily V member 4	F: AGAAAGCGCCCATGGATT
	R: TCTGTGGCTGCTTCTCTACG
MDR1 Multidrug resistance protein 1	F: TGTTCAAACTTCTGCTCCTGA
	R: CCCATCATTGCAATAGCAGG
ABCC1 ATP-binding cassette sub-family C member 1	F: GGAAGTAGGGCCCAAAGGTC
	R: AGGACACGTCGGAACAAGTC

11 $${\text{Relative gene expression}=2}^{-\Delta \Delta \text{ct}}$$

#### Reactive oxygen species (ROS)

The levels of Reactive Oxygen Species Modulator 1 (ROS Modulator 1) were measured using a Rat ROS Modulator 1 ELISA Kit (Catalog No: E1924r, Nova Lifetech) via a sandwich ELISA technique. Cell lysates were prepared by rinsing adherent cells with pre-cooled PBS, trypsinization, and centrifugation. Cells were washed, suspended in PBS, and subjected to multiple freeze–thaw cycles. The supernatant, obtained after centrifugation, was used for analysis. In the assay, 100 μL of standards, blanks, and samples were added to wells and incubated at 37 °C for 2 h. Detection Reagent A and B were sequentially applied with intervening washes, followed by TMB substrate addition for color development. The reaction was stopped with sulfuric acid, and absorbance was read at 450 nm to quantify ROS Modulator 1 levels. Each treatment was performed in triplicate.

### Statistical analysis

An analysis of nanoparticle size distribution was conducted using ImageJ software, employing mean and standard deviation calculations to determine central tendency and variability. Supplemented by ocular examination using the “Histogram” feature. Three independent repetitions of the experiments were did, and the IC_50_ results were assessed with GraphPad Prism 8.0. Presented were the values of mean and standard deviation. An analysis of variance (ANOVA) with post hoc Tukey’s analysis, performed using SPSS program (IBM Corp., USA) version 20, identified statistically significant variations in gene expression among the groups. Statistical significance was defined as a value of P < 0.05. To explore the relationships between gene expression patterns, Pearson’s correlation coefficients were calculated and visualized using Python’s seaborn library for data visualization. This approach provides an intuitive overview of how strongly and in what direction gene expression levels are associated across different treatment groups. Heatmaps were generated to highlight both positive and negative correlations, offering insights into potential co-regulation or opposing gene activity in response to experimental conditions.

## Results and discussion

### Ultraviolet measurements (UV)

**Fig. 1:**
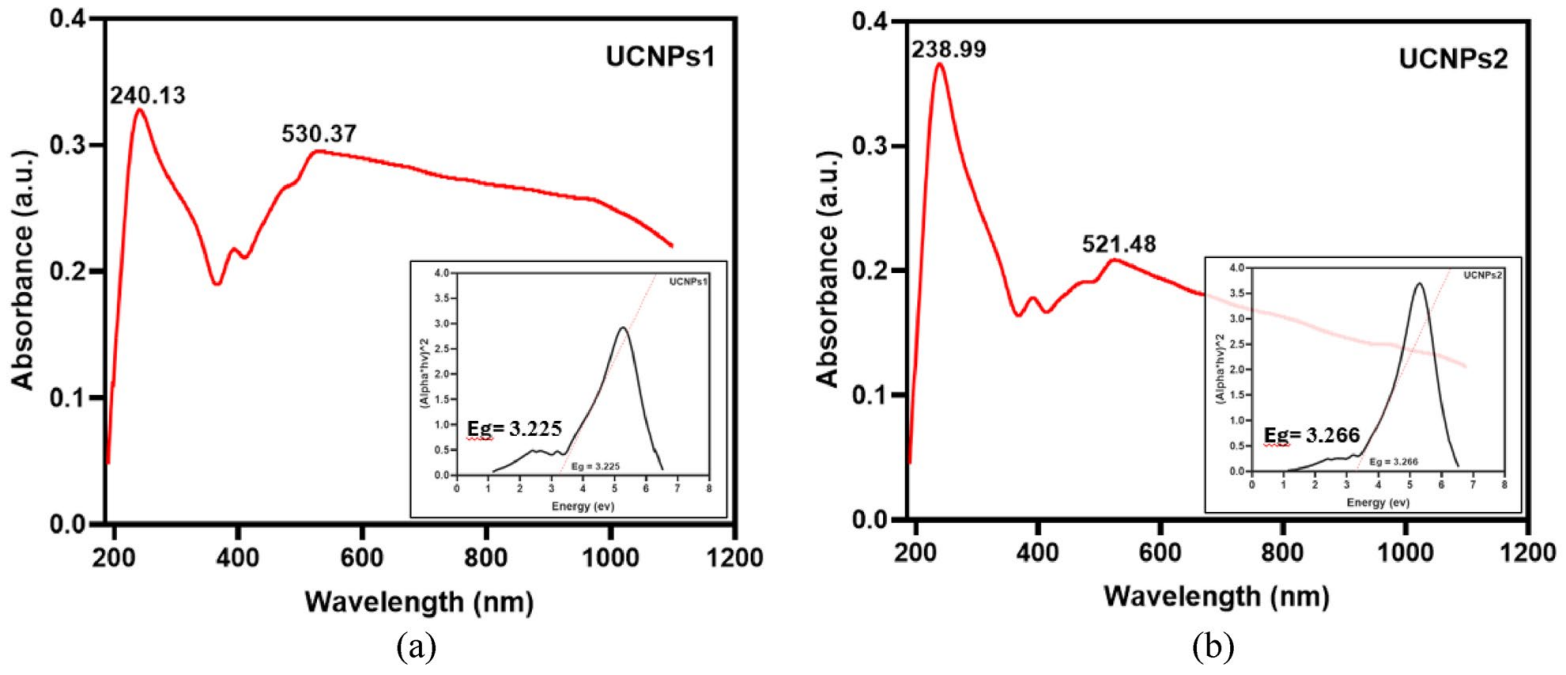
UV–VIS absorption spectra and energy gap of the (**a**) UCNPs1, and (**b**) UCNPs2.

The UV–Vis absorption spectra of UCNPs1 and UCNPs2 were recorded to analyze their optical properties. For UCNPs1, two distinct absorption peaks were observed at 240 nm and 530 nm, indicating strong absorption in both the UV and visible regions. In contrast, UCNPs2 exhibited absorption peaks at 283 nm and 521 nm, reflecting slight shifts in both the UV and visible absorption compared to UCNPs1 due to variations in the concentrations of rare earth metals used during the synthesis of the nanoparticles^71^. The characteristic peaks observed in the spectra are indicative of the UCNPs’ optical properties. The optical absorption corresponding to the 6H5/2 → 4G5/2 transition of Sm^3^⁺ ions was noted around 480–550 nm^72^. While our UCNPs showed an emission peak around 530.37 and 521.48 nm (a wavelength within the visible range known to have biological effects) we did not use this wavelength directly. Visible light, including 530 and 521.48 nm, has limited ability to penetrate biological tissues, which restricts its effectiveness in deeper regions. To overcome this, we used a 980 nm near-infrared (NIR) laser for excitation. NIR light is widely recognized for its deeper tissue penetration and lower scattering, making it more appropriate for biomedical applications. The UCNPs absorb the NIR light and then emit light at shorter wavelengths, such as visible or UV, allowing us to deliver biologically active light more effectively to targeted internal tissues. The UCNPs1 exhibits a direct bandgap of approximately 3.225 eV, while UCNPs2 shows a slightly higher value of around 3.266 eV shown in Fig. 1. These bandgap values indicate that both nanoparticles are suitable for applications requiring efficient absorption and emission of light in the UV–visible range^52^.

### X-ray photoelectron spectroscopy (XPS)

**Fig. 2:**
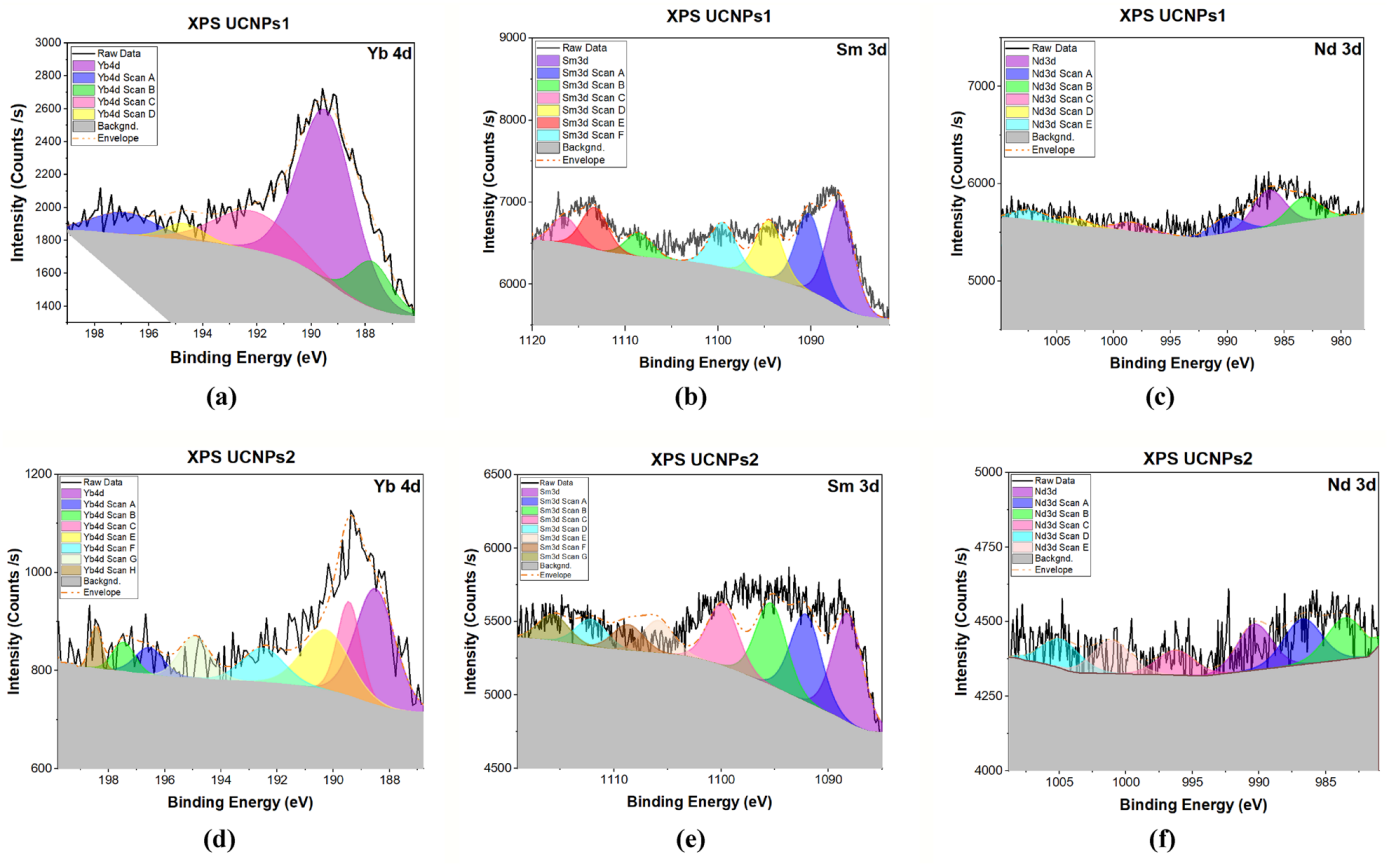
High-resolution XPS core-level spectra of a UCNPs1 and UCNPs2 corresponding to (**a**, **d**) Yb-4d, (**b**, **e**) Sm-3d and (**c**, **f**) Nd-3d. They are deconvoluted into Gaussian peaks.

Figure 2a,b investigates the oxidation states of Yb in the UCNPs1 and UCNPs2 samples, for UCNPs1 the Yb 4d XPS spectrum was carefully deconvoluted. The resulting peaks indicated the presence of both Yb^3^⁺ and Yb^2^⁺ oxidation states. Peaks observed at approximately 187.76, 192.18, and 194.7 eV are attributed to Yb^3^⁺, while the peaks at 189.45 and 196.9 eV correspond to the 4d₃/₂ and 4d₅/₂ components of Yb^2^⁺, respectively. This distribution confirms the coexistence of mixed valence states within the UCNPs1 structure. Similarly, the Yb 4d XPS spectrum of the UCNPs2, sample was deconvoluted to identify the oxidation states of Yb. The peaks at 192.5, 194.69, 196.55, and 197.46 eV are assigned to Yb^3^⁺, while those appearing at approximately 188.51 and 189.25 eV are associated with the 4d₅/₂ and 4d₃/₂ components of Yb^2^⁺. The nearby signal at 190.03 eV may represent overlapping contributions from both oxidation states. This complex spectral pattern, with partially merged features, further supports the presence of a mixed-valence environment in UCNPs2^73–75^.

Figure 2b,e investigates the oxidation states of Sm in the UCNPs1 and UCNPs2 samples, for UCNPs1 the Sm 3d XPS spectrum was carefully deconvoluted. The resulting peaks revealed the coexistence of both Sm^3^⁺ and Sm^2^⁺ species. Notably, peaks at approximately 1086.8 eV and 1108.5 eV correspond to the spin–orbit components of Sm^3^⁺ (3d₅/₂ and 3d₃/₂), while peaks at 1090.2 eV and 1113.3 eV are attributed to Sm^2^⁺. Additional features detected at 1094.5, 1099.5, and 1116.5 eV may be linked to satellite peaks or Auger-related transitions^65^, commonly observed in rare-earth oxide systems. Similarly, the Sm 3d XPS spectrum of the UCNPs2 sample was also deconvoluted to assess the distribution of oxidation states. Peaks located at approximately 1088.2 eV and 1112.1 eV are attributed to the Sm^3^⁺ 3d₅/₂ and 3d₃/₂ components, while the peaks at 1091.5 eV and 1115.7 eV indicate the presence of Sm^2^⁺. Additional peaks at 1094.6, 1098.0, 1101.5, and 1106.8 eV may correspond to satellite features or Auger transitions typically observed in mixed-valence systems^66,67^.

Figure 2c,f explores the chemical state of Nd in the synthesized UCNPs1 and UCNPs2 samples, X-ray photoelectron spectroscopy (XPS) was performed, focusing on the Nd 3d region. The spectra revealed two distinct spin–orbit doublets corresponding to the Nd 3d₅/₂ and Nd 3d₃/₂ levels, which are characteristic of trivalent neodymium (Nd^3^⁺). In the case of UCNPs1, the 3d₅/₂ and 3d₃/₂ components are observed at approximately 986.3 eV and 1007.3 eV, respectively, with an energy separation of ~ 21 eV^76^, consistent with values reported for Nd^3^⁺ in neodymium oxide environments. Additional peaks at 983.3 eV and 1003.5 eV may reflect the presence of mixed chemical environments, including contributions from Nd–O bonds and potential shake-up satellites^77^. Similarly, the UCNPs2 sample displays a comparable spin–orbit splitting pattern, with peaks at 985.5 eV (3d₅/₂) and 1007.9 eV (3d₃/₂), suggesting that neodymium remains predominantly in the Nd^3^⁺ state. Subtle features around 982.5 eV and 996.2 eV may be associated with surface-related states or partial electron transfer involving the 4f orbitals. Overall, the XPS results confirm that Nd exists primarily as Nd^3^⁺ in both samples, with minor variations possibly arising from local structural or coordination differences^78,79^.

### X-ray diffraction (XRD)

**Fig. 3:**
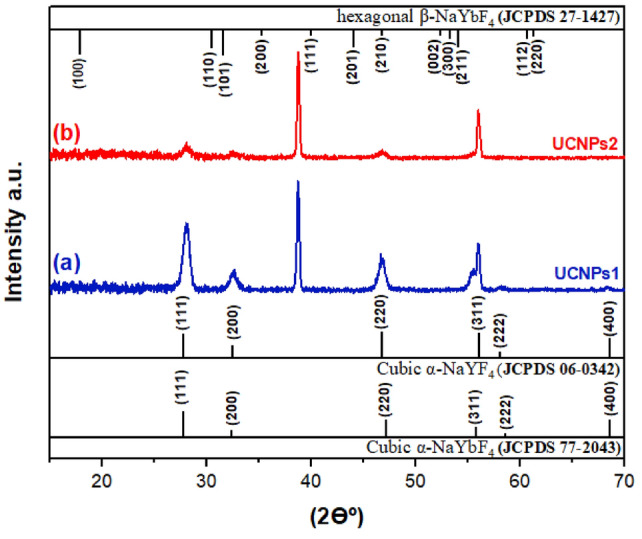
The x-ray diffraction (XRD) pattern result of the NaYbF_4_:Sm,Nd, the space structure and phase component of the synthesized UCNPs.

Using X-ray diffraction (XRD), the space structure and phase component of the synthesized UCNPs were clarified (Fig. 3). The α-NaYbF_4_ is the isostructure to the α-NaYF_4_ (JCPDS 06-0342). The standard pattern (JCPDS 27- 1427) of β-NaYbF_4_ is shown in Fig. 3, all the observed diffraction peaks closely matched those of hexagonal β-NaYbF_4_ (JCPDS 27-1427) and cubic α-NaYbF_4_ (JCPDS 77-2043) and cubic α-NaYF_4_ (JCPDS 06-0342). Additionally, there were no secondary phase impurity peaks, indicating that the resulting UCNPs had hybrid cubic and hexagonal phase. NaREF_4_ can exist in either the cubic phase, which is a metastable high-temperature form, or the hexagonal phase, which is the thermodynamically stable low-temperature form^34,80^. Table 2 presents a comparative analysis of two upconversion nanoparticle samples, UCNPs1 and UCNPs2, highlighting differences in lattice parameters, crystal size, degree of crystallinity, strain, and particle size. The interplanar spacing (d_hkl) is nearly identical for both samples, indicating a similar crystal structure. However, the lattice parameter "a" is slightly larger in UCNPs2 (6.356 Å) than in UCNPs1 (6.331 Å), which suggests minor lattice expansion possibly due to strain where UCNPs1 is (0.00185 ± 0.00108) and UCNPs2 is (0.00288 ± 0.00249). The c-axis parameter remains nearly the same, reflecting structural stability. The crystal size, calculated from XRD, is 22.63 nm for UCNPs1 and 33.39 nm for UCNPs2, whereas the TEM-derived particle size is 11 nm for UCNPs1 and 8 nm for UCNPs2. Furthermore, the Degree of Crystallinity (Table 2) of the synthesized upconversion nanoparticles was evaluated, the ratio of the integrated area of crystalline peaks to the total area under both crystalline and amorphous peaks in the XRD pattern^81^. Based on this approach, UCNPs1 exhibits a much higher degree of crystallinity (91.29%) compared to UCNPs2 (56.06%), implying that UCNPs1 has fewer structural defects, which is beneficial for optical properties. The differences in crystallinity between the two samples could be attributed to variation in dopant concentrations.

**Table 2:** Interplanar spacing, lattice parameters, crystal size, degree of crystallinity, and strain of UCNPs1 and UCNPs2, obtained from XRD and TEM analysis.

Sample	Interplanar spacing	Lattice parameters		Crystal size (nm)	Degree of crystallinity (%)	Strain	Particle size (TEM)
	${d}_{hkl}$ Å	“a” Å	“c” Å				(nm)
UCNPs1	2.323	6.331 Å	2.450 Å	22.631	91.291	0.00185 ± 0.00108	11
UCNPs2	2.322	6.356 Å	2.448 Å	33.395	56.057	0.00288 ± 0.00249	8

### Fourier transform infrared (FTIR)

**Fig. 4:**
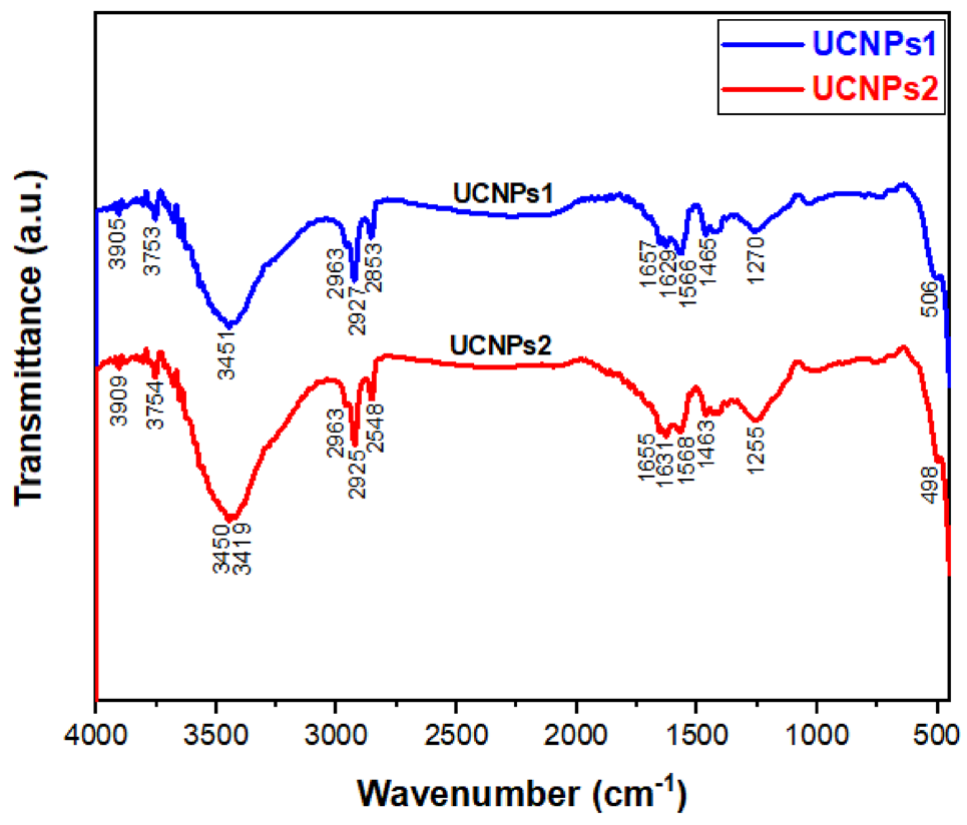
FT-IR spectrum for UCNPs1 and UCNPs2.

The FTIR spectrum of the NaYbF_4_:Sm^3+^,Nd^3+^ UCNPs was obtained to investigate the surface ligands of the resulting samples, and the corresponding results are displayed in Fig. 4. It Shows no difference in the FT-IR results between the two UCNPs. For UCNPs1 the broad absorption band around 3451 cm^−1^ corresponds to the stretching vibration of –O–H due to the presence of oleic acid. The FT-IR peaks at 1465 and 2927 cm^−1^ are attributed to the stretching vibration of -C-H. The FT-IR peaks at 1657 cm^-1^ are attributed to the stretching vibration of –C=C– stretch alkenes. For UCNPs2 the broad absorption band around 3450 cm^−1^ corresponds to the stretching vibration of –O–H due to the presence of oleic acid. The FT-IR peaks at 1463 and 2925 cm^−1^ are attributed to the stretching vibration of –C–H. The FT-IR peaks at 1655 cm^-1^ are attributed to the stretching vibration of –C=C–^82^.

### Transmission electron microscopy (TEM)

**Fig. 5:**
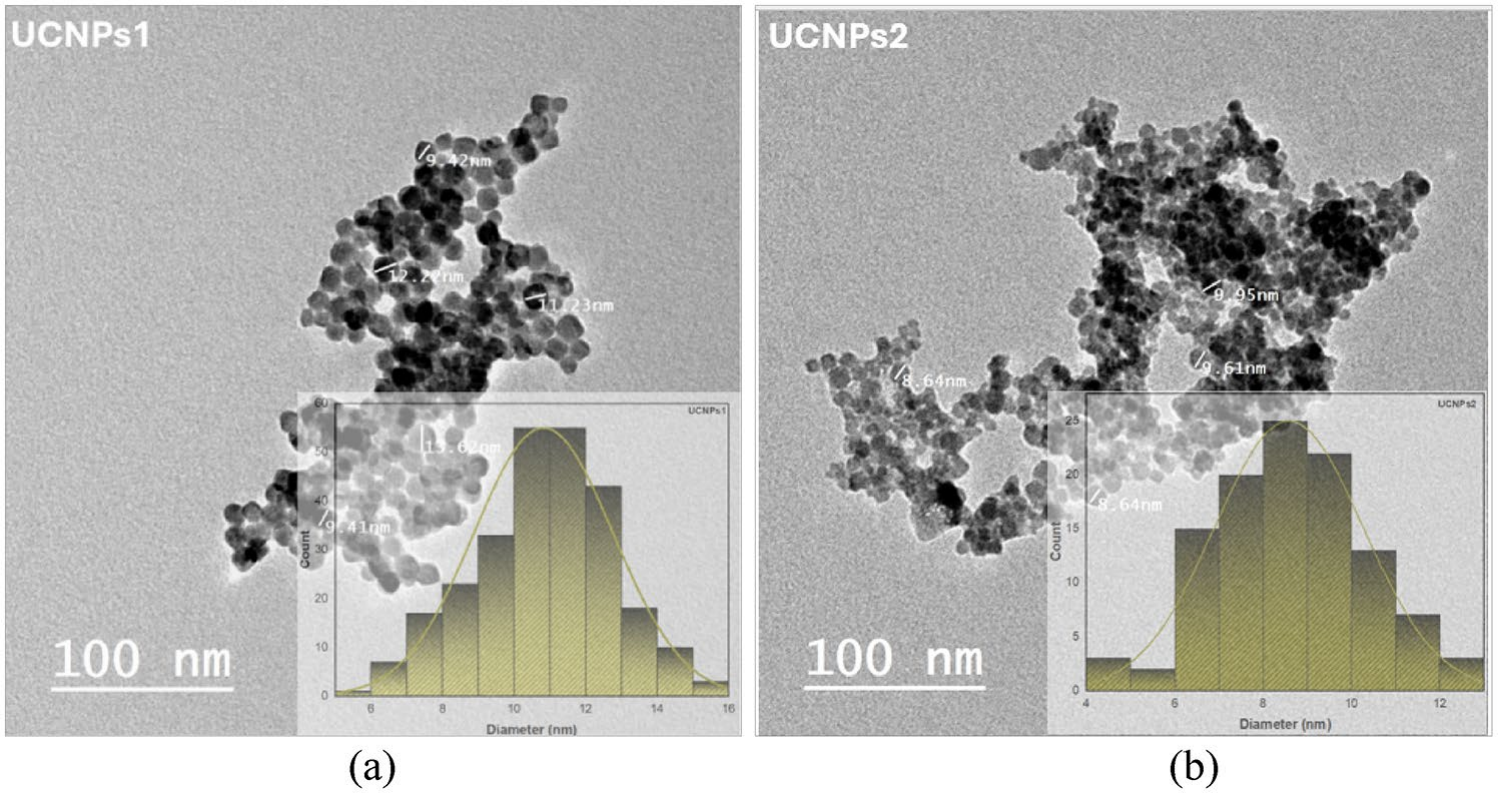
TEM images of UCNPs (**a**) for NaYbF_4_:Sm,Nd with concentrations (75, 20, 6 mol%) and (**b**) for NaYbF_4_:Sm,Nd with concentrations (80, 15, 5 mol%). Showing the size distribution of each UCNPs confirming the successful synthesis of uniformly nanocrystalline.

The size and morphology of the synthesized NaYbF_4_:Sm,Nd UCNPs were evaluated by TEM, as shown in Fig. 5. It showed that NaYbF_4_:Sm,Nd UCNPs formed in the nanoscale and spherical shape, where the particle size average between 8 to 12 nm (Table 2).

**Fig. 6:**
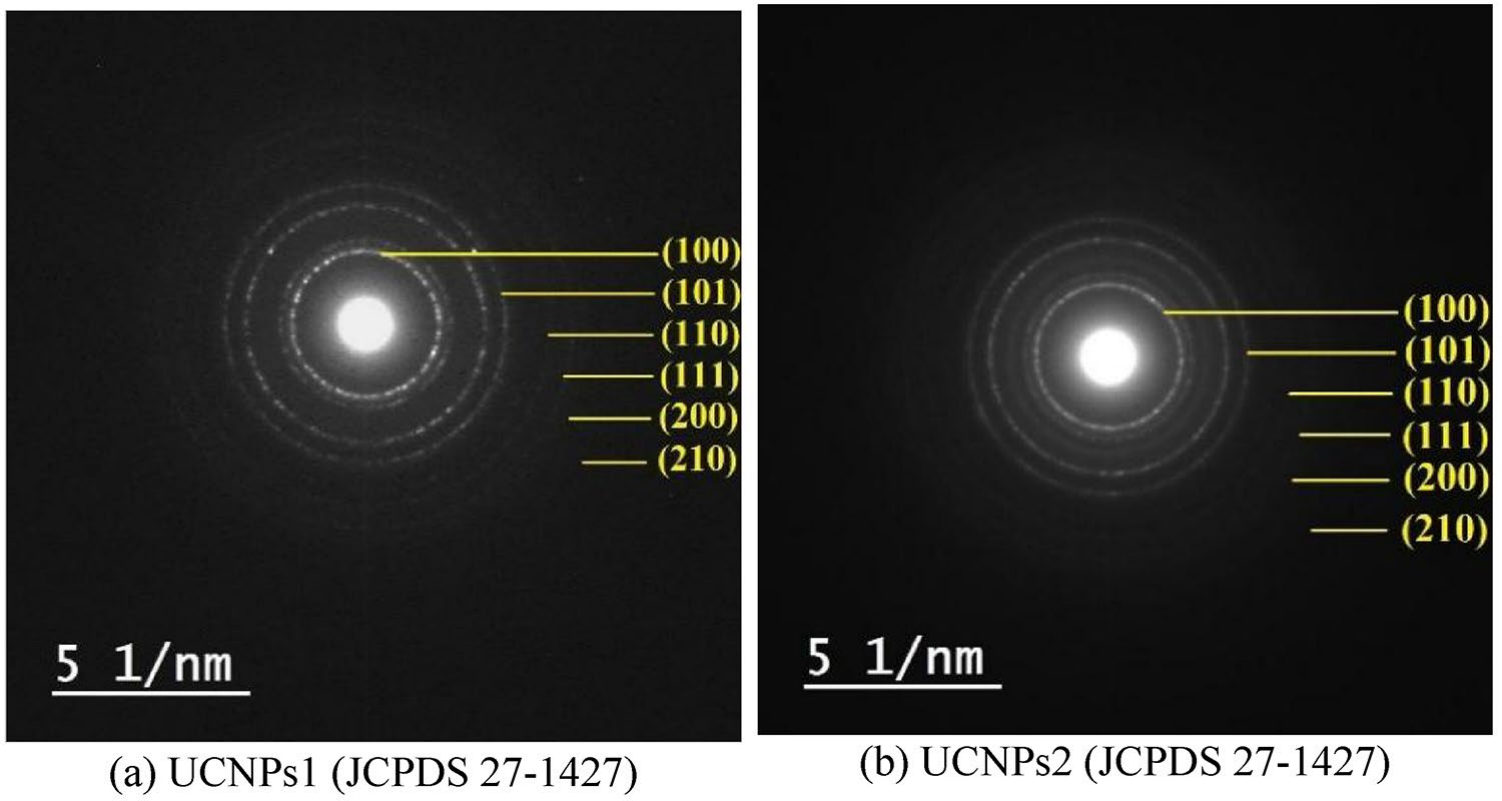
The diffraction rings (**a**) for UCNPs1 (NaYbF₄:Sm,Nd; 75, 20, 6 mol%) and (**b**) for UCNPs2 (NaYbF₄:Sm,Nd; 80, 15, 5 mol%) are identified using JCPDS card (27–1427) with Miller indices marked by arrows.

The electron diffraction pattern Fig. 6 consisting of rings reveals the good crystal structure of the nanoparticles. Patterns correspond to the crystal field (110), (101), (111) and (201)^83^, which is in agreement with XRD data.The XRD patterns revealed that the UCNPs adopted mixed cubic and hexagonal phases, consistent with the selected synthesis conditions. These structural features were corroborated by TEM in close agreement with the crystallite sizes estimated from XRD. The SAED diffraction rings further supported the high crystallinity of the samples, aligning well with the XRD peak assignments.

### Dispersive X-ray analysis (EDX)

The EDX analysis revealed the presence of all elements in the nanocrystals, including the doping lanthanides Yb, Sm, and Nd. The weight and atomic ratios of Yb, Sm, and Nd are shown in Table 3, which was close to the stoichiometric ratio of the reactants used in the experiment.

**Table 3:** EDX weight ratio of UCNPs using two spectrums with different concentrations.

UCNPs	Ytterbium (Yb)		Samarium (Sm)		Neodymium (Nd)		Sodium (Na)		Fluoride (F)		Oxygen (O)	
	Weight (%)	Atomic (%)	Weight (%)	Atomic (%)	Weight (%)	Atomic (%)	Weight (%)	Atomic (%)	Weight (%)	Atomic (%)	Weight (%)	Atomic (%)
UCNPs1	13.94	1.68	1.71	0.24	0.59	0.09	23.32	21.19	43.51	47.85	1.12	1.46
UCNPs2	20.50	0.21	0.33	0.04	0.10	0.01	27.63	21.44	45.47	42.70	1.80	2.01

**Fig. 7:**
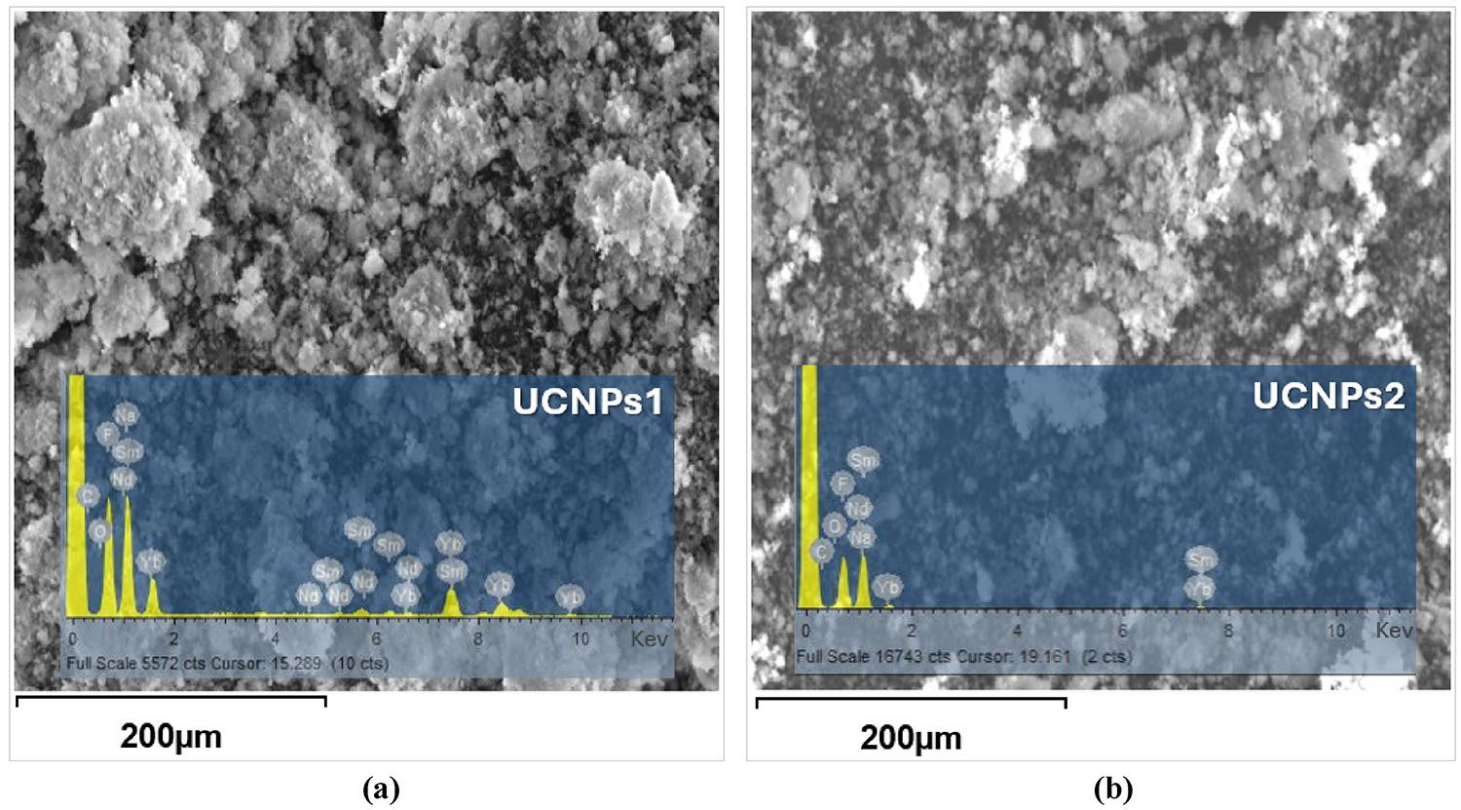
EDX analysis confirms the UCNPs (NaYbF₄:Sm, Nd) composite formation. Spectra (**a**) and (**b**) shows the elemental composition of the synthesized nanostructure, revealing the presence of Ytterbium (Yb), Samarium (Sm), Neodymium (Nd), Sodium (Na), Fluoride (F), and Oxygen (O).

In order to confirm the formation of the UCNPs (NaYbF_4_:Sm,Nd) composite EDX analysis was conducted. Various areas of the sample were examined during the EDX measurement, and the corresponding peaks are depicted in Fig. 7a,b. The EDX spectrum of the synthesized composite nanostructure reveals the presence of Ytterbium, Samarium, Neodymium, Sodium, Fluoride, and Oxygen. In spectrum (a), the values of Yb, Sm, Nd, Na, F and O in atomic % were 1.68, 0.24, 0.09, 21.19, 47.85, 1.46 , respectively, while in spectrum (b), the values were 0.21, 0.04, 0.01, 21.44, 42.70, 2.01 measured in atomic % for Yb, Sm, Nd, Na, F and O respectively. Details of all EDX spectra for the UCNPs, including values measured in both atomic and weight percentages, are listed in Table 3. XPS and EDX provided complementary insights into the chemical composition and oxidation states of the lanthanide ions. While EDX confirmed the stoichiometric incorporation of Yb, Sm, and Nd into the NaYbF₄ host lattice, XPS analysis verified the predominance of trivalent oxidation states (Yb^3^⁺, Sm^3^⁺, Nd^3^⁺). This evidence establishes the chemical environment required for efficient energy transfer among dopants, supporting the observed optical behavior.

### Surface roughness and waviness

**Fig. 8:**
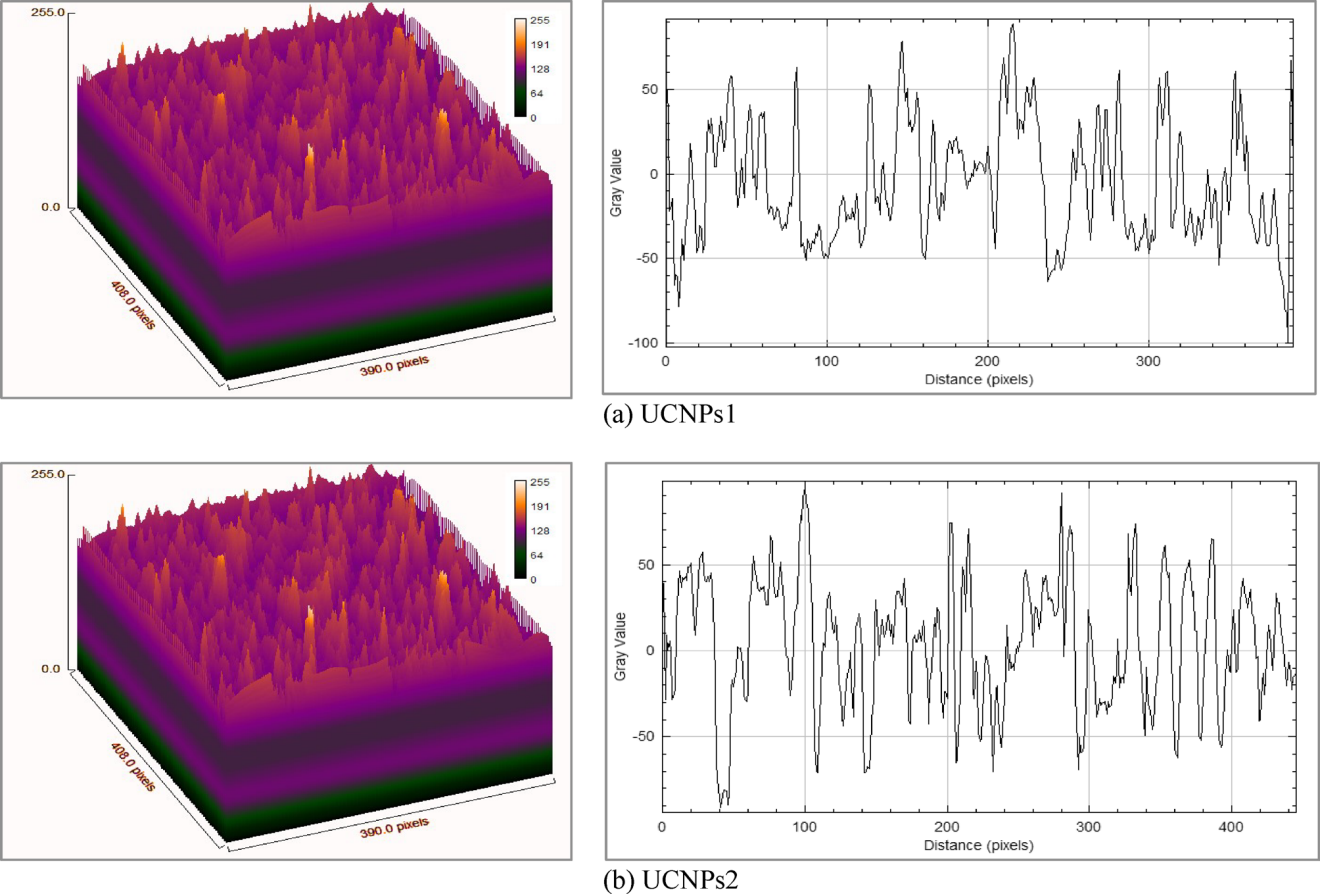
The 3-D and 2-D surface profiles representation for (**a**) UCNPs1, (**b**) UCNPs2. The figures provide a detailed visualization of the surface characteristics, UCNPs1 exhibits a roughness of 35.5142 nm, while UCNPs2 shows 40.7221 nm roughness. Differences in the concentration of rare-earth metals in UCNPs1 and UCNPs2 led to variations in their observed roughness scales. The analysis was conducted using ImageJ.

**Fig. 9:**
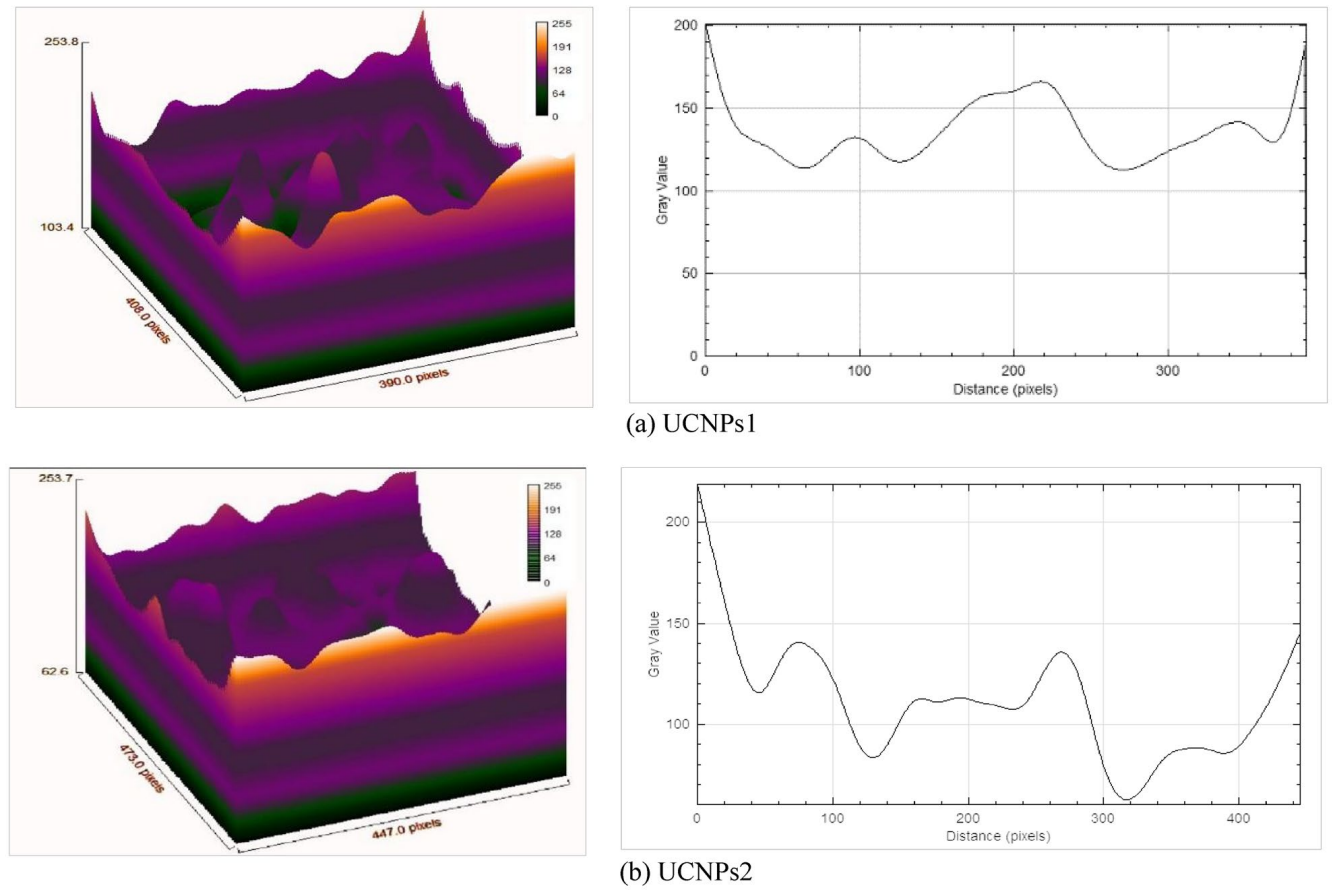
Waviness profiles for (**a**) UPCNs1 and (**b**) UPCNs2 slices. The graph illustrates the differences in waviness parameters, including root mean square waviness (Rq), average waviness (Ra), and total waviness height (Rt), highlighting the surface texture variations between the two slices.

Figure 8a,b display the surface topological profiles and roughness curves for UCNPs1 and UCNPs2. The analysis of the roughness and waviness data for UPCN1 and UPCN2 slices reveals notable differences across several surface characteristics. As shown in Figs. 8 and 9 and detailed in Table 4, roughness parameters such as root mean square roughness (Rq) and average roughness (Ra) are higher for UCNPs2, indicating a rougher and more irregular surface compared to UCNPs1. Roughness Skewness (Rsk) and kurtosis (RKu) values indicate variations in surface asymmetry and peak sharpness as detailed in Table 4. Surface texture analysis, based on roughness parameters, provides quantitative insights into deviations from the mean profile. Key parameters include Ra, which represents the mean roughness; Rq, indicating root mean square deviation of the surface profile from the mean line; Rsk quantifies profile asymmetry, while RKu describes the sharpness of peaks and valleys. UCNPs2 exhibits greater maximum valley depth (Rv) and peak height (Rp), resulting in a significantly higher total profile height (Rt), as shown in Fig. 8.

**Table 4:** Roughness and waviness parameters for UCNPs1 & UCNPs2.

Slice	Rq	Ra	Rsk	RKu	Rv	Rp	Rt	Rc	FPO	MFOV	FAD	MRV	SA
Roughness UCNPs1	35.5142	28.1027	0.5229	0.3352	− 103.7153	130.3047	234.0200	1.5963	79.2966	76.7992	0.6406	0.0699	11.5464
Roughness UCNPs2	40.7221	32.6702	− 0.1664	− 0.0637	− 166.4990	126.6819	293.1810	0.3492	80.9916	81.5176	3.9130	0.0982	14.0868
Waviness UCNPs1	22.0256	17.3913	1.1553	0.9436	− 35.0899	76.9436	112.0335	− 2.2759	44.9786	51.6056	152.7459	0.0616	1.8513
Waviness UCNPs2	22.0354	17.3446	0.5037	0.4976	− 51.4266	74.4216	125.8482	− 6.3597	46.8348	56.2586	32.6382	0.0298	1.8093

Waviness data further reveal similarities in root mean square waviness (Rq) and average waviness (Ra) between both samples, with UCNPs2 displaying a slightly higher total waviness height (Rt), suggesting marginally more pronounced surface features (Table 4). Additional parameters, including the mean height of the profile elements (Rc), frequency of profile occurrences (FPO), mean frequency of occurrences (MFOV), frequency amplitude distribution (FAD), mean radius of valleys (MRV), and surface area (SA) also show variations highlighting differences in the surface texture and structure between the slices (Fig. 9).

### Zeta potential measurements

**Fig. 10:**
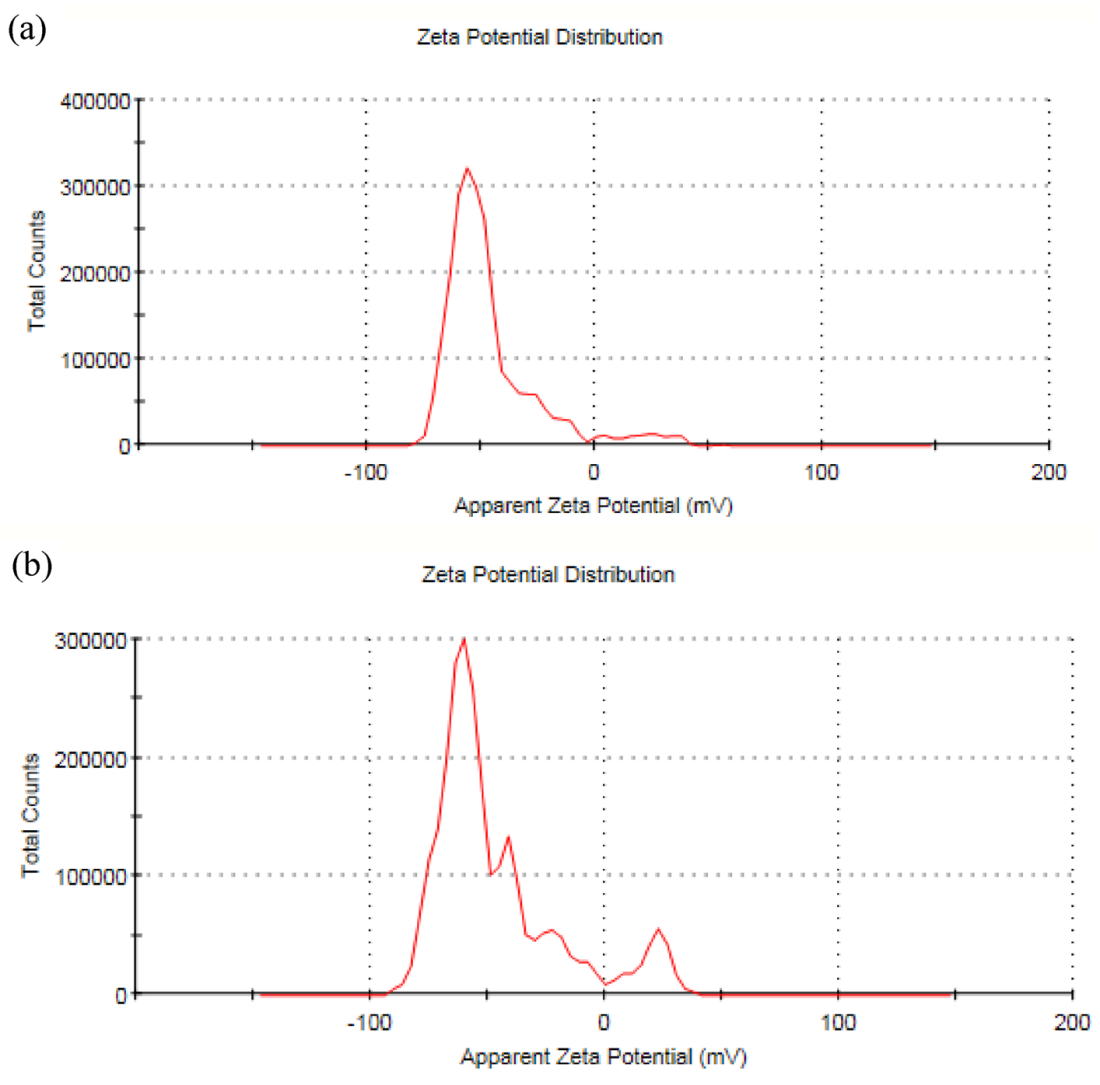
Zeta potential measurement of (**a**) UCNPs1 and (**b**) UCNPs2, indicating surface charge and colloidal stability in the phosphate buffer saline (PBS, 0.01 M, pH 7.4).

Zeta potential of UCNPs1 exhibited 46 ± 14 mV and UCNPs2 exhibited 46.4 ± 8.48 mV are shown in Fig. 10a,b respectively. This significant zeta potential indicates relatively high negative charge density on the surface of nanoparticles because of the generation of repulsive forces between nanoparticles. Nanoparticles with a zeta potential more negative than -30 mV or more positive than + 30 mV are generally considered stable .^84^ For intracellular theranostics, it’s essential to have monodispersed particles with a small size and uniform shape to ensure consistent optical properties, efficient cellular uptake, and predictable biological effects^85,86^.

**Fig. 11:**
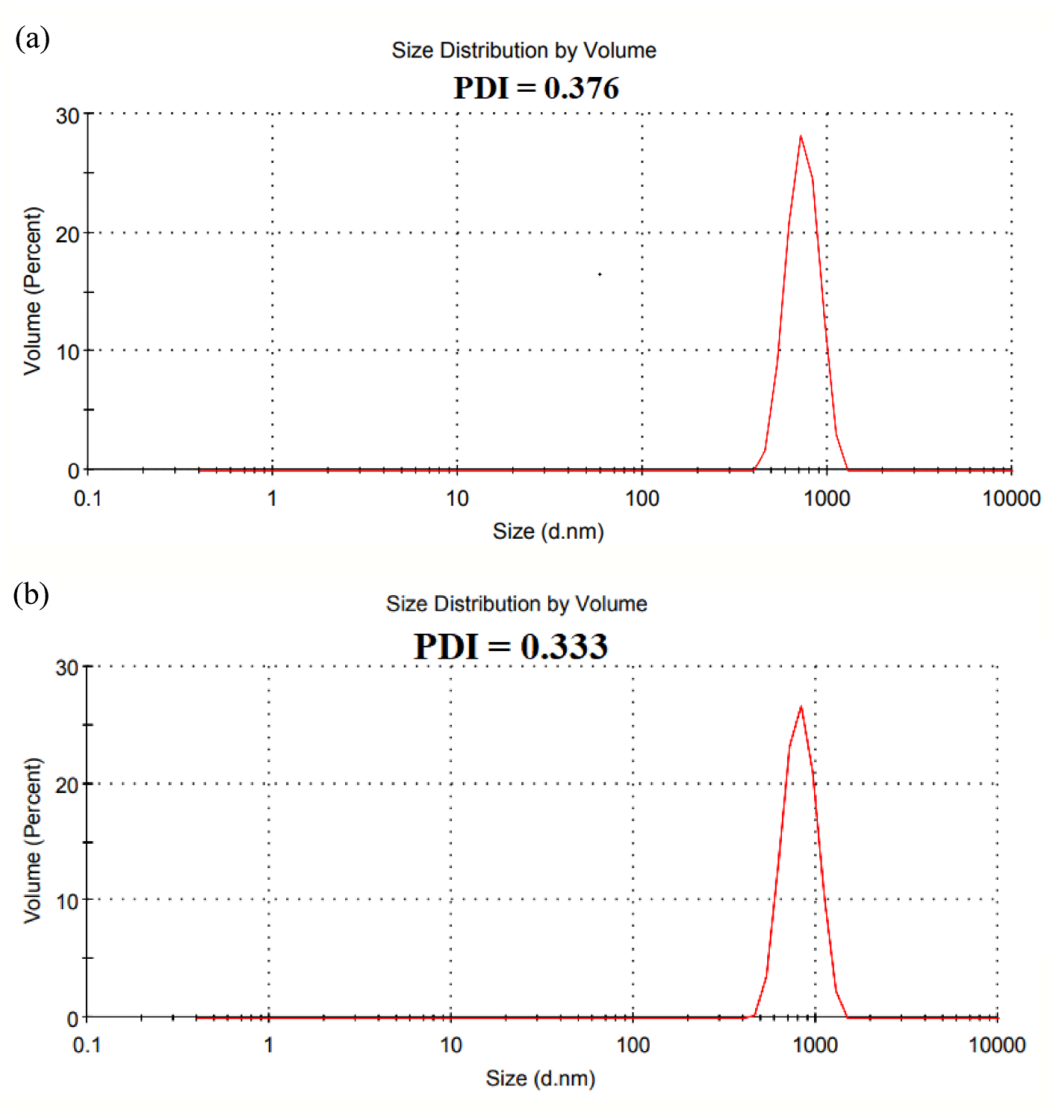
DLS analysis of (**a**) UCNPs1 and (**b**) UCNPs2, illustrating the size distribution and polydispersity index of the nanoparticles.

The average size of UCNPs1 (Z-Average 1027 d.nm) and UCNPs2 (Z-Average 1044 d.nm) obtained by Dynamic Light Scattering Measurements (DLS) as shown in Fig. 11 is higher than that observed in TEM measurements, possibly due to non-uniform size distribution and aggregation of UCNPs in phosphate buffer saline (PBS, 0.01 M, pH 7.4). Where the Polydispersity Index (PDI) for both UCNPs is 0.3 which indicates a monodisperse distribution^87,88^. Surface roughness, zeta potential, and DLS measurements provided a detailed picture of nanoparticle stability in suspension. The high absolute zeta potential values (> 30 mV) indicated strong electrostatic repulsion, minimizing aggregation. Although DLS showed slightly larger hydrodynamic diameters compared to TEM, this is consistent with surface ligand effects and the aqueous environment. In addition that the DLS data represent a size distribution by volume. Together with surface roughness analysis, these results highlight the nanoparticles’ stable dispersion and well-defined morpholog.

### Photo luminance

**Fig. 12:**
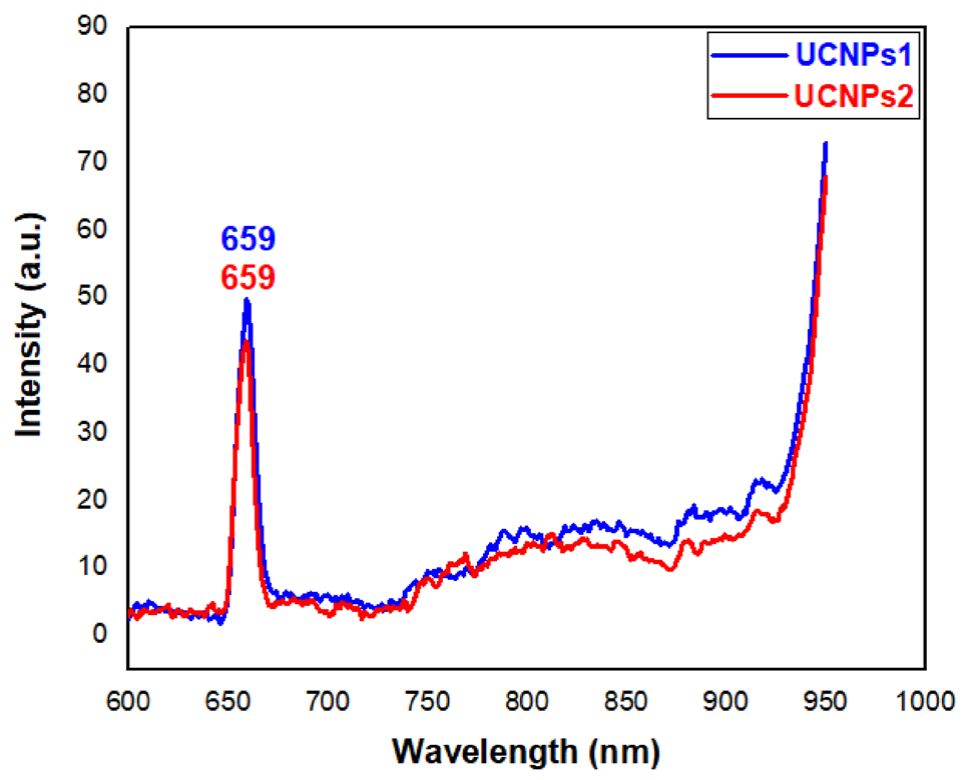
PL of UCNPs1 and UCNPs2 showed red emission (659 nm) under 980 nm excitation, UCNPs1 showed higher emission intensity than UCNPs2.

The UCL spectra for the NaYbF_4_:Sm,Nd UCNPs shown in Fig. 12, exhibit red emission at 659 nm under 980 nm excitation with a power density of 0.022 W/cm^2^. Then with some modification in the concentrations of the doping materials we found that the intensity of red upconversion emission was improved for UCNPs1 than UCNPs2 that may be attributed to doped concentration or cubic α-phase to Hexagonal β-phase phase transition as shown in Fig. 12^7,89,90^. Lattice strain plays a significant role in determining material properties, with UCNPs2 exhibiting a higher strain (0.00288 ± 0.00249) than UCNPs1 (0.00185 ± 0.00108). This increased strain in UCNPs2 leads to more lattice defects and non-radiative energy loss, negatively impacting its luminescence efficiency. The combination of higher crystallinity, lower strain, and a well-defined lattice structure in UCNPs1 explains why it exhibits superior fluorescence. Conversely, UCNPs2’s lower crystallinity and higher strain contribute to a more defect-rich structure, reducing its optical performance.

When UCNPs excited by an appropriate light source such as near-infrared (NIR) radiation, exhibit unique optical properties and energy conversion capabilities. The process begins with sensitization by the sensitizer ions, which efficiently absorb NIR photons due to their electronic energy levels. The absorbed photons elevate the energy levels of the sensitizer ions, which then transfer this energy to the activator ions through cross-relaxation, where the energy of an excited sensitizer ion is transferred to a neighboring activator ion. This energy transfer promotes the activator ions to higher energy levels within their respective electronic structures. Upon relaxation back to their ground states, the activator ions emit photons in the visible or ultraviolet range through multi-photon processes, resulting in emitted photons with higher energies than the originally absorbed NIR photons^91,92^.

The mechanism of energy absorption, transfer, and emission in the upconversion process for UCNPs involving Yb^3^⁺ as the sensitizer, Nd^3^⁺ as intermediate activator, and Sm^3^⁺ as activator or emitter, can be explained as follows. Yb^3^⁺ absorbs 980 nm NIR light and transitions from the ^2^F_7/2_​ level to the ^2^F_5/2_ level^93^. The excited ^2^F_5/2_​ state of Yb^3^⁺ transfers energy to Nd^3^⁺. Nd^3^⁺ then transfers energy to Sm^3^⁺. Sm^3^⁺ can be directly excited by Yb^3^⁺. Transitions from ^4^G_5/2_ level to ^6^H_9/2_ level​ of Sm^3^⁺ result in visible light emission at 659 nm^94^. This energy transfer results in distinct emission wavelengths, characteristic of the Sm^3^⁺ activator, corresponding to its respective energy transition. This effectively demonstrates the role of Yb^3^⁺ in sensitizing the UCNPs, enabling the upconversion process that leads to the emission of higher-energy photons^95^.

XPS analysis (Fig. 2) confirms the presence of Yb^3^⁺, Nd^3^⁺, and Sm^3^⁺ in their trivalent states, which are essential for the sequential energy transfer processes in upconversion nanoparticles. These results align well with the expected electron transitions: Yb^3^⁺ serves as the main NIR photon absorber, Nd^3^⁺ facilitates energy transfer to Yb^3^⁺, and Sm^3^⁺ acts as the final emitter. The identification of these oxidation states supports the proposed energy migration pathway within the UCNPs, indicating that the observed chemical environment is consistent with efficient light absorption, transfer, and emission^96^. PL measurements were directly correlated with the structural and compositional data. The superior red emission intensity observed for UCNPs1 can be attributed to its higher crystallinity, lower lattice strain, and optimal dopant incorporation, as confirmed by XRD, TEM, and XPS. In contrast, the weaker emission of UCNPs2 is explained by its lower crystallinity and higher structural strain, which favor non-radiative relaxation pathways.

### Hemolysis assay

**Fig. 13:**
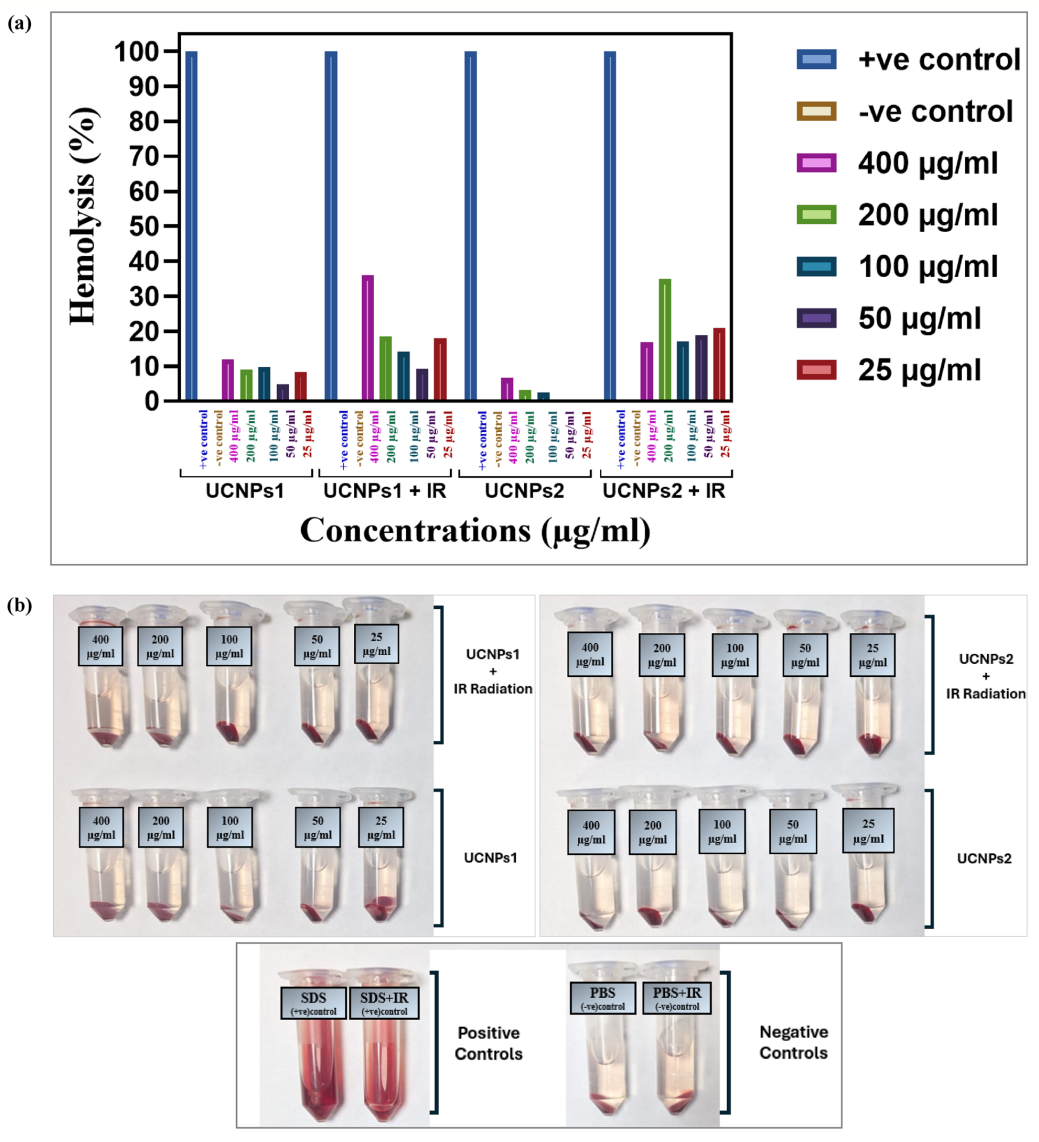
(**a**, **b**) illustrate the hemolytic effect on red blood cells (RBCs) following three hours of incubation with UCNPs1 and UCNPs2, both in the presence and absence of infrared (IR) irradiation at varying concentrations (400–25 µg/mL). Phosphate-buffered saline (PBS) was employed as the negative control to represent normal conditions, while sodium dodecyl sulfate (SDS) served as the positive control to induce complete hemolysis.

The hemolysis assay conducted on UCNPs1, UCNPs2, UCNPs1 + IR and UCNPs2 + IR at concentrations ranging from 400 to 25 µg/mL revealed concentration-dependent effects on red blood cells (RBCs) Fig. 13a,b. Phosphate-buffered saline (PBS) served as the negative control, showing minimal hemolysis, while sodium dodecyl sulfate (SDS) acted as the positive control, representing 100% hemolysis. Both UCNPs1 and UCNPs2 exhibited increased hemolysis at higher concentrations. While the combination of UCNPs1 or UCNPs2 with infrared (IR) irradiation resulted in more pronounced hemolytic activity compared to treatment without IR.

**Fig. 14:**
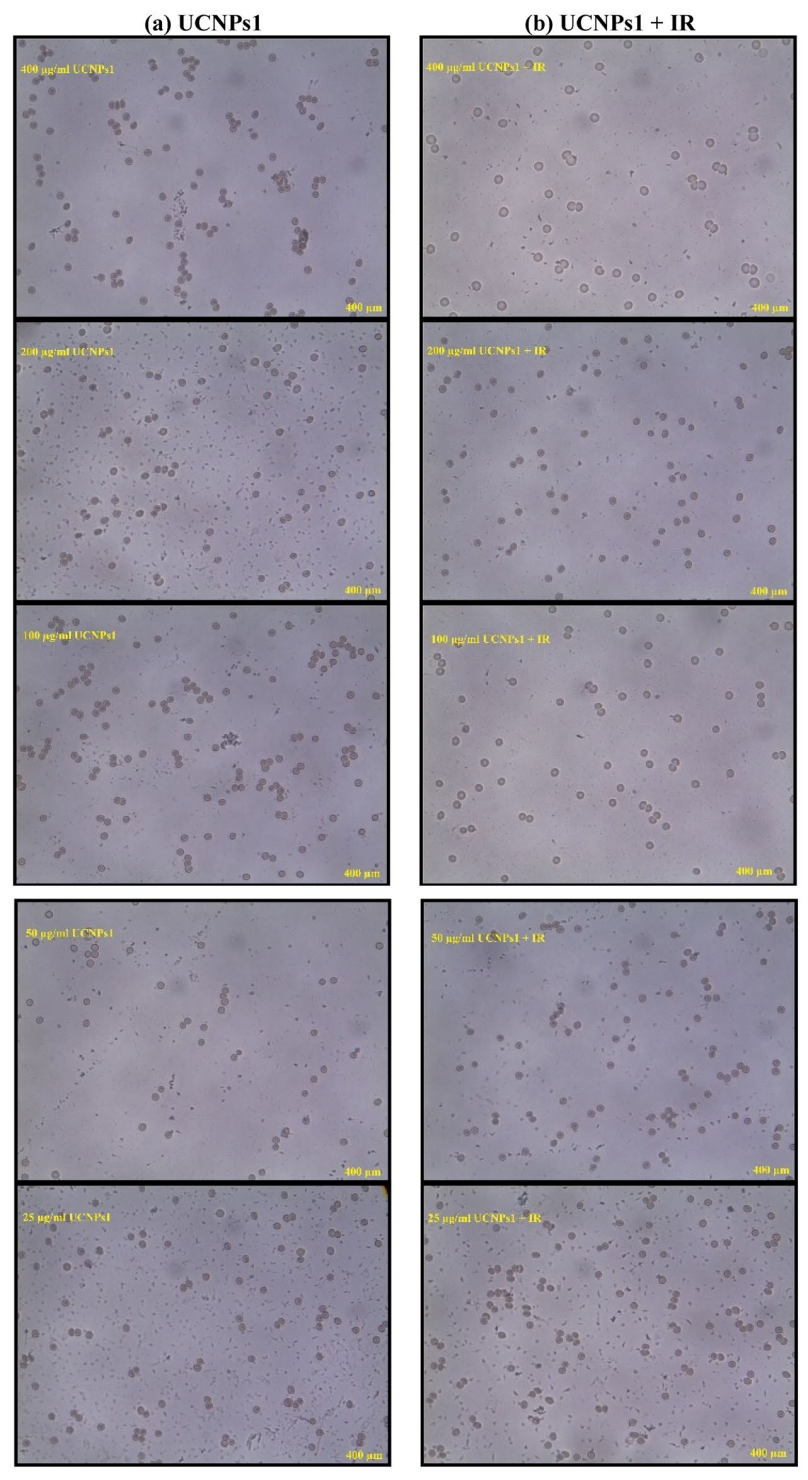
Display microscopic images of the RBCs post treatment with different concentrations of (**a**) UCNPs1 (**b**) UCNPs1 + IR showing morphological changes.

**Fig. 15:**
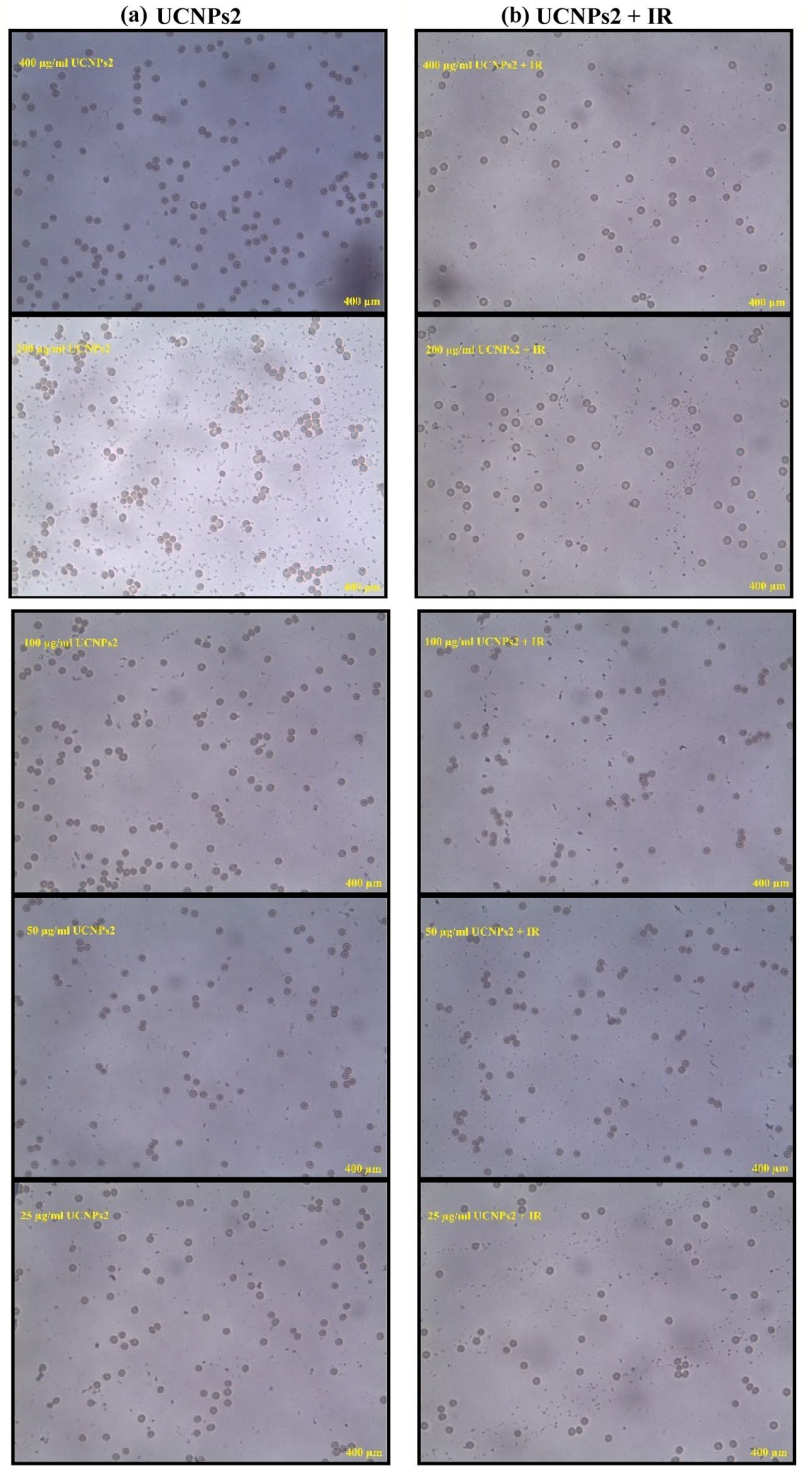
Display microscopic images of the RBCs post treatment with different concentrations of (**a**) UCNPs2 (**b**) UCNPs2 + IR showing morphological changes.

**Fig. 16:**
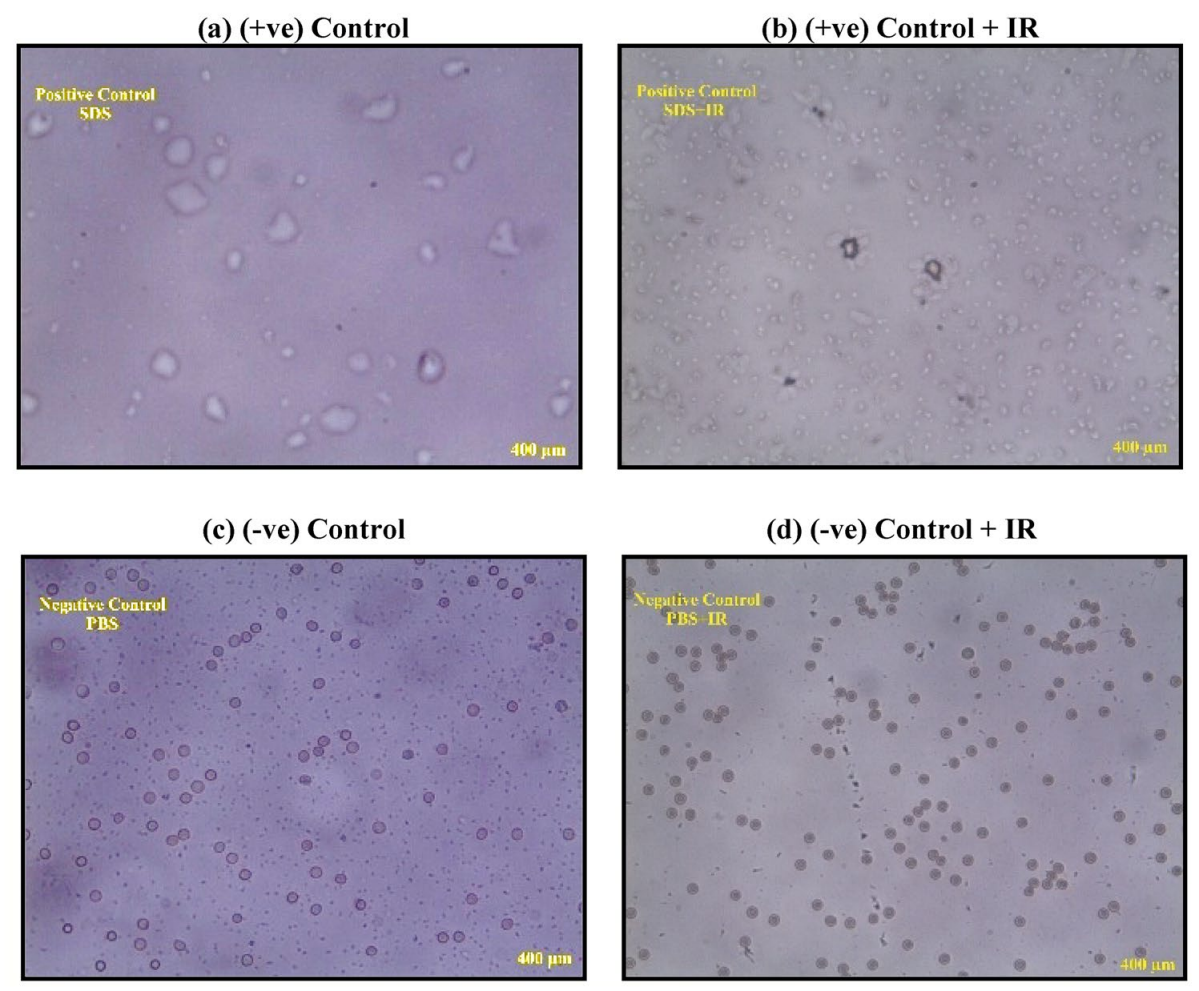
Display microscopic images of the RBCs (**a**) (+ ve) Control, (**b**) (+ ve) Control + IR, (**c**) (−ve) Control, and (**d**) (−ve) Control + IR showing morphological changes.

The microscopic images in Figs. 14, 15, 16 illustrate the morphological response of RBCs under different treatments. In Fig. 14, cells treated with increasing concentrations of UCNPs1 show slight structural changes, yet hemolysis remains below 10%, indicating limited cytotoxicity. When irradiation (IR) is applied in combination with UCNPs1, the images reveal more pronounced alterations in cell shape and integrity, reflecting an increase in hemolysis. Figure 15 shows the effect of UCNPs2, where the images demonstrate fewer visible changes compared to UCNPs1, confirming its lower hemolytic potential. However, with the addition of IR, the RBCs exposed to UCNPs2 display a similar degree of damage as those treated with UCNPs1 plus IR, suggesting that irradiation exerts a comparable influence regardless of nanoparticle type. The control images in Fig. 16 further validate these observations: positive control and positive control + IR samples exhibit extensive damage and loss of normal structure, while negative control and negative control + IR samples preserve the characteristic biconcave morphology. Taken together, the sequence of images highlights that while UCNPs1 induce slightly greater hemolysis than UCNPs2 under normal conditions, IR exposure equalizes their damaging effects on RBCs.

### In vitro cytotoxicity and cell viability assays (MTT assay)

**Fig. 17:**
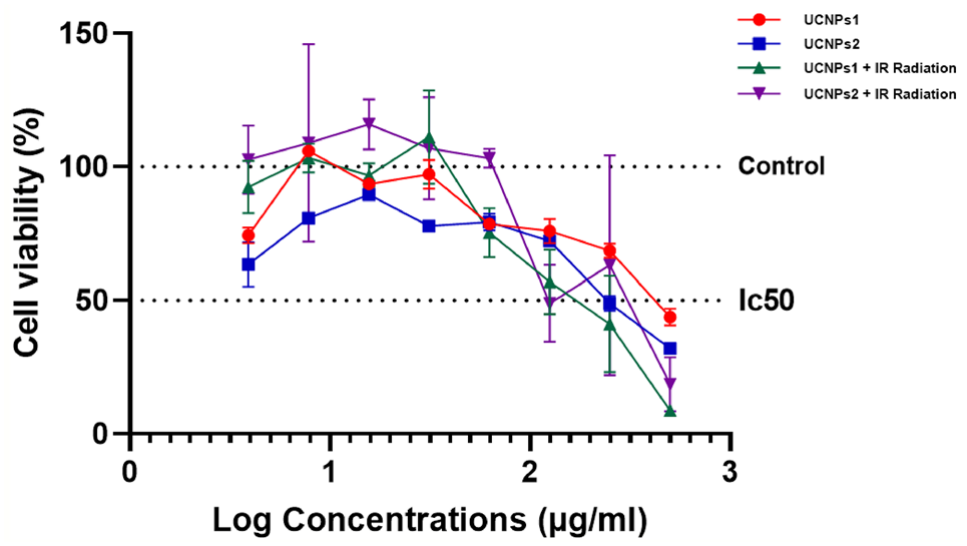
IC50 curves show the 50% inhibitory concentrations (IC50) of UCNPs1, UCNPs2, UCNPs1 + IR, UCNPs2 + IR.

In an MTT assay, the IC50 values for irradiated UCNPs 1 and 2 were found to be 165.9 µg/ml and 237.9 µg/ml, respectively. These values are significantly lower than those obtained from the MTT assay for non-irradiated UCNPs 1 and 2, which were 460.7 µg/ml and 372.3 µg/ml, respectively as shown in Fig. 17. Northby that control cell that not irradiated and irradiated has no significant change in viability. Notably, the untreated (control) cells and the cells exposed to radiation alone showed no significant change in viability. This indicates that UCNPs absorb NIR light, transfer energy from sensitizer to activator ions, and emit higher-energy visible or UV photons through multi-photon processes. When high-energy photons, such as those from ultraviolet (UV) light, interact with biological molecules, can generate reactive oxygen species (ROS), which can overwhelm antioxidant defenses and cause oxidative stress. This imbalance leads to cellular damage, including lipid peroxidation, DNA fragmentation, and apoptosis. UV radiation induces ROS through direct interaction with cellular components or via photosensitization. It also affects enzymatic pathways, such as catalase inhibition, nitric oxide synthase (NOS) upregulation, and reduced protein kinase C (PKC) expression, further increasing ROS levels. The extent of ROS-induced damage depends on the cell’s intracellular oxidative state^97^.

### Wound healing after drug and IR irradiation

**Fig. 18:**
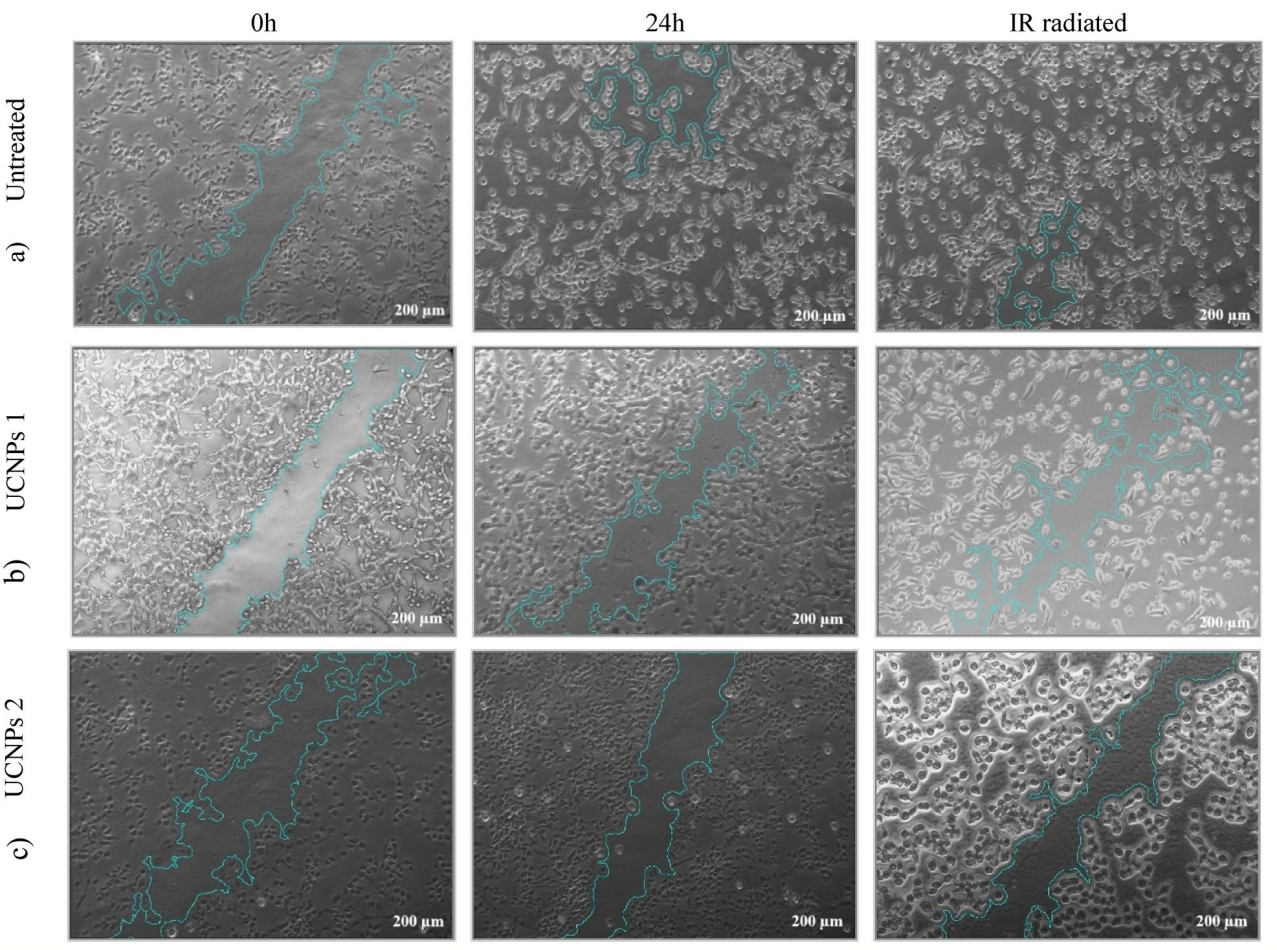
Wound healing assay showing the migration of T24 cell line over 48 h under different treatment conditions. Representative images were taken at 0h, 24h after scratch formation and 24h after radiation. (**a**) Untreated (Control). (**b**) Cells treated with UCNPs1 in combination with radiation. (**c**) Cells treated with UCNPs2 in combination with radiation. The calculations were performed using ImageJ software. Images obtained by inverted-light optical microscope with magnification at 200 μm (20×).

The wound healing assays were performed at the IC₅₀ concentrations of UCNPs1, UCNPs2, radiated UCNPs1, and radiated UCNPs2 as established by the preceding MTT viability test. Data reveals significant differences in wound closure rates between the untreated (control) group and groups treated with UCNPs1, UCNPs2, radiation only (radiated control), radiated UCNPs1, and radiated UCNPs2 (Fig. 18). The untreated group demonstrated vigorous wound healing, with 62.72% closure, while the Radiation only group showed an enhanced closure rate of 79.32%, indicating that radiation positively influences tissue repair in untreated cells (Table 5).

**Table 5:** Wound healing assay results: wound closure rates for untreated (control), UCNPs1, and UCNPs2 groups with and without radiation treatment.

Sample	Wound area T0	Wound area T24	Wound area T48 with IR	% Wound closure without IR	% Wound closure with IR
Untreated (control)	333,824 ± 119.182	124,443 ± 153.176	69,019 ± 75.602	62.72%	79.32%
UCNPs 1	293,475 ± 53.002	248,429 ± 93.796	253,278 ± 174.995	15.35%	13.70%
UCNPs 2	333,587 ± 108.316	260,670 ± 53.554	273,764 ± 112.325	21.86%	17.93%

In contrast, UCNPs1 and UCNPs2 exhibited markedly reduced wound closure rates. UCNPs1 showed only 15.35% closure without radiation and 13.70% with radiation, while UCNPs2 achieved 21.86% closure without radiation and 17.93% with radiation. These results suggest that both nanoparticles impair wound healing, with UCNPs1 having a more pronounced inhibitory effect. Furthermore, radiation worsened the impaired healing in nanoparticle-treated groups, indicating a potential interference with cellular repair mechanisms.

### Bladder (T24) matrix proteins

Analytical tools for studying protein–protein (PPI) and chemico-protein interactions using computational methods.

**Fig. 19:**
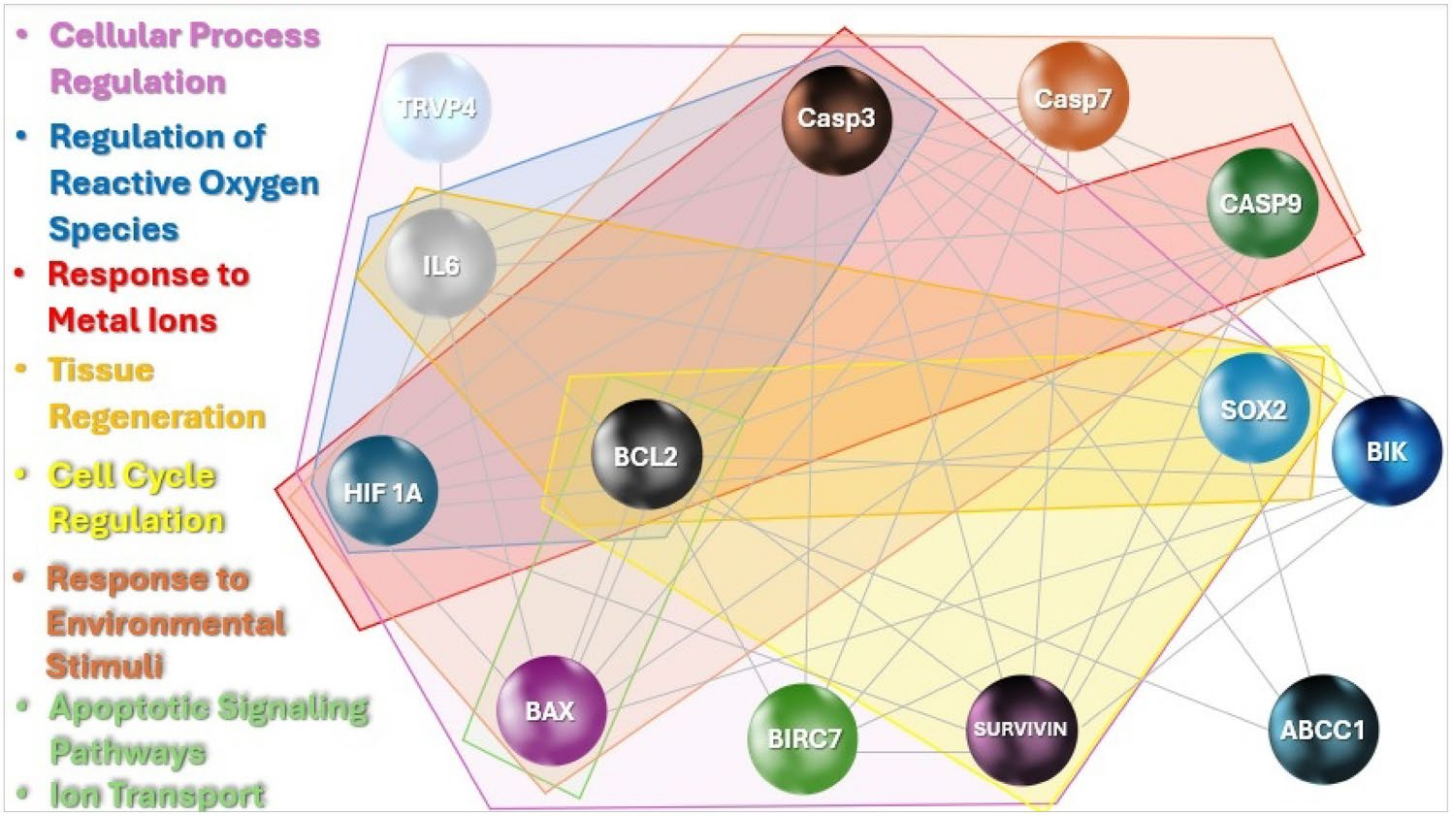
PPI network analyzed by bioinformatics: identification of key interacting proteins involved in cellular processes, apoptotic signaling pathways, ion transport, regulation of reactive oxygen species, environmental responses, metal ion reactions, tissue regeneration, and cell cycle regulation.

The network (Fig. 19) consists of 17 nodes and 43 edges, with an average of 5.059 neighbors per node. This moderate degree of connectivity suggests that the network is fairly well connected. The network’s diameter is 4, meaning the longest shortest path between any two nodes is just 4 steps, and its radius is 2, indicating that the network’s center is only 2 steps away from the farthest node. The characteristic path length is 1.860, demonstrating that communication within the network is efficient, with relatively short paths between nodes. The clustering coefficient of 0.595 suggests moderate clustering, indicating the presence of local groups or modules within the network. With a density of 0.316, the network has a relatively low number of edges compared to the total possible connections, but there is still a significant level of interaction. Network heterogeneity is 0.472, showing a moderate variability in node connectivity, where some nodes are more highly connected than others. The network has a centralization value of 0.421, indicating that it is moderately centralized around key nodes. Importantly, the network consists of a single connected component, meaning all nodes are part of one large interconnected structure. The analysis was completed quickly, with a computation time of just 0.033 s, highlighting the efficiency of the process.

### Conventional polymerase chain reaction (cPCR)

**Fig. 20:**
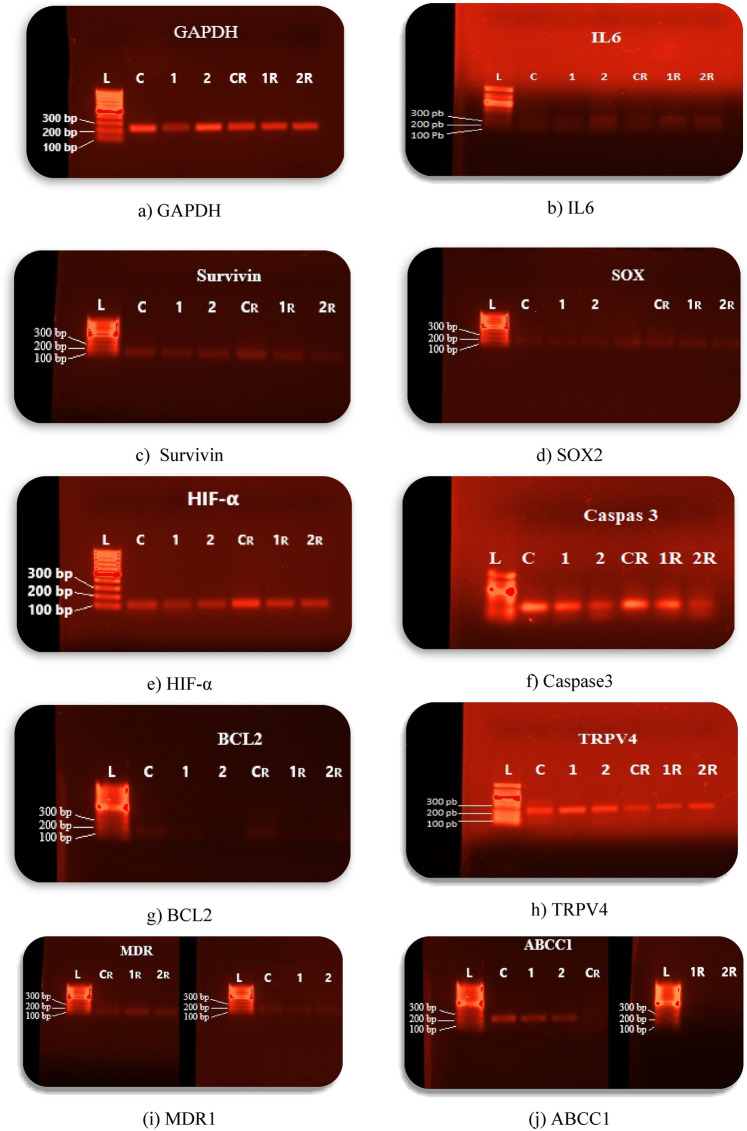
Gel Electrophoresis of Gene Expression in T24 Cancer Cells Following Treatment with UCNPs1, UCNPs2, and Radiation, The gel image shows PCR amplification products for GAPDH (internal control), IL-6, SURVIVIN, SOX2, HIF-α, Caspase 3, BCL2, TRPV4, MDR1, and ABCC1. The gel lanes are designated as follows: (L) molecular weight ladder, (C) untreated (control), (CR) Radiation only (radiated control), (1) UCNPs1, (2) UCNPs2, (1R) radiated UCNPs1, and (2R) radiated UCNPs2.

The gel electrophoresis results reveal significant variations in the expression of key genes SOX2, Survivin, BCL2, IL-6, TRPV4, and Caspase 3 in T24 cancer cells treated with *the IC₅₀ concentrations of each treatment group (UCNPs1, UCNPs2, radiated UCNPs1, and radiated UCNPs2) as established by the preceding MTT viability test..* Differential band intensities across treatment groups (untreated (control), UCNPs1, UCNPs2, Radiation only (radiated control), *radiated UCNPs1, and radiated UCNPs2*)indicate that UCNPs and radiation modulate critical cellular pathways as shown in Fig. 20. Further quantification by RT-PCR will be conducted to validate these observations and explore their implications for targeted cancer therapy.

### Real-time quantitative PCR (RT-PCR)

**Fig. 21:**
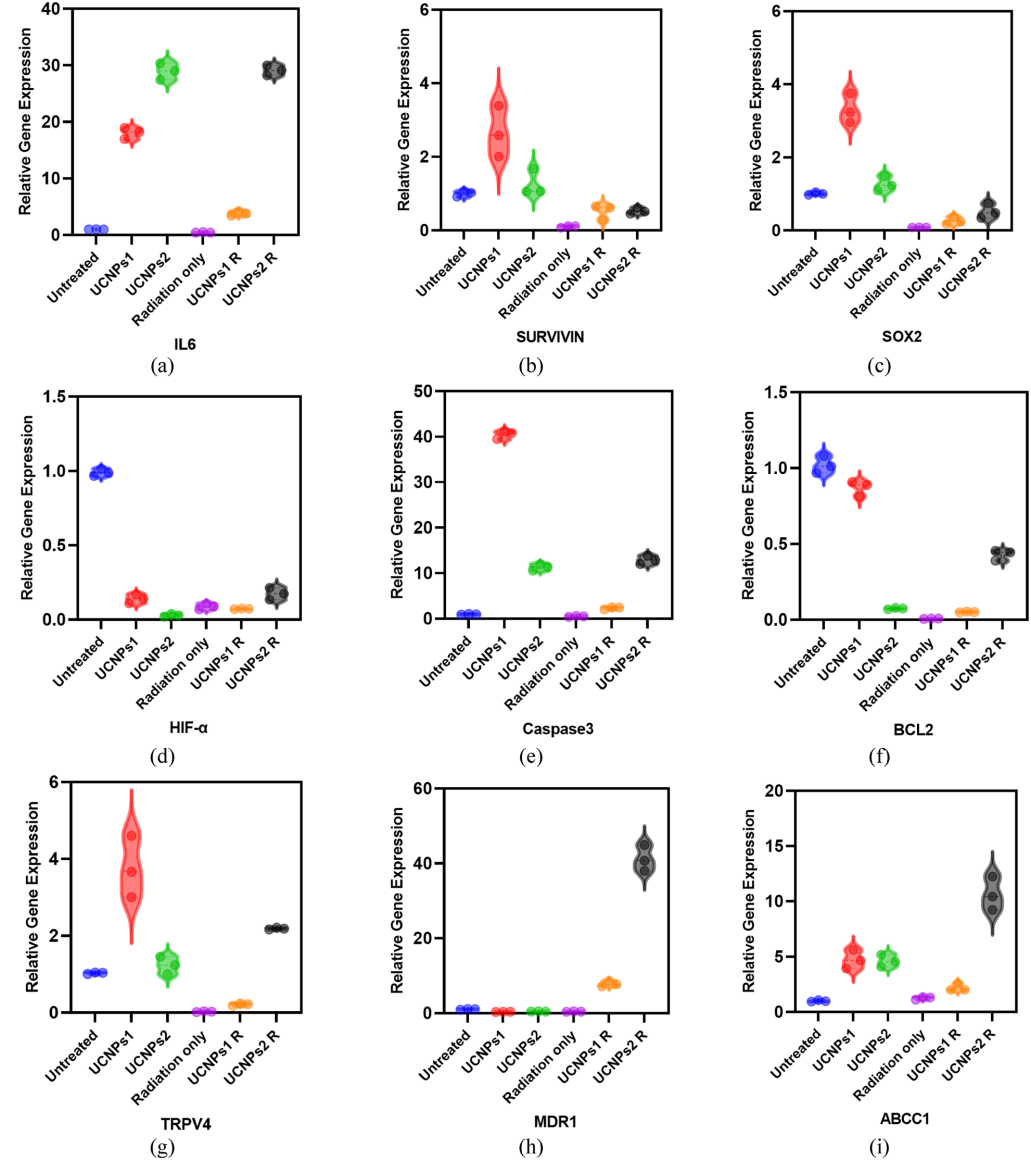
Superplot of Relative gene expression levels under different treatment conditions. Plots show the expression of: (**a**) IL-6, (**b**) SURVIVIN, (**c**) SOX2, (**d**) HIF-α, (**e**) Caspase 3, (**f**) BCL2, (**g**) TRPV4. (**h**) MDR1, and (**i**) ABCC. Gene expression levels are shown as fold changes relative to untreated control, normalized to GAPDH by the 2^–ΔΔCt^ method.

The data Table 6 and Fig. 21 presented reveals the impact of the IC50 concentrations of different treatments (untreated (control), UCNPs1, UCNPs2, Radiation only (radiated control), radiated UCNPs1, and radiated UCNPs2) on the expression of several key genes related to inflammation, stem cell properties, and apoptosis in cancer cells. The data presented in Table 6 highlights the differential effects of UCNPs1 and UCNPs2 on various biological markers, both before and after radiation exposure. UCNPs2 exhibited a stronger inflammatory response, as indicated by higher IL6 levels compared to UCNPs1. Interestingly, while radiation significantly reduced IL6 levels in the untreated (control) and UCNPs1 groups, the expression remained elevated in the radiated UCNPs2 group, suggesting a sustained inflammatory effect.

**Table 6:** Comparison of gene expression in bladder (T24) cells treated with UCNPs1, UCNPs2, Radiated UCNPs1 and Radiated UCNPs2, Values represent relative gene expression (fold changes) normalized to GAPDH using the 2^–ΔΔCt^ method. Untreated control values were set to 1.0.

	Untreated	UCNPs1	UCNPs2	Radiation only	Radiated UCNPs1	Radiated UCNPs2
IL6	0.99 ± 0.01	18.05 ± 1*	28.96 ± 1.5*^#^	0.45 ± 0.05^#$^	3.81 ± 0.4*^#$@^	29.1 ± 0.9*^#@&^
SURVIVIN	1 ± 0.08	2.66 ± 0.7*	1.26 ± 0.4^#^	0.1 ± 0.03^#$^	0.51 ± 0.2^#^	0.52 ± 0.1^#^
SOX2	1.01 ± 0.036	3.32 ± 0.4*	1.27 ± 0.2^#^	0.087 ± 0.006*^#$^	0.266 ± 0.1*^#$^	0.52 ± 0.2^#$^
HIF-α	0.99 ± 0.02	0.13 ± 0.03*	0.02 ± 0.009*^#^	0.08 ± 0.02*	0.07 ± 0.002*	0.17 ± 0.04*^$@&^
Caspase3	0.99 ± 0.07	40.5 ± 0.9*	11.3 ± 0.7*^#^	0.51 ± 0.1^#$^	2.329 ± 0.2^#$@^	12.91 ± 0.9*^#@&^
BCL2	1.02 ± 0.05	0.87 ± 0.05*	0.07 ± 0.004*^#^	0.007 ± 0.001*^#^	0.05 ± 0.002*^#^	0.42 ± 0.03*^#$@&^
TRPV4	1.0 ± 0.02	3.75 ± 0.8*	1.23 ± 0.2^#^	0.02 ± 0.007*^#$^	0.21 ± 0.03^#$^	2.18 ± 0.02*^#$@&^
MDR1	1.03 ± 0.09	0.35 ± 0.03	0.45 ± 0.05	0.42 ± 0.03	7.81 ± 0.71*^#$@^	41.21 ± 3.47*^#$@&^
ABCC1	0.99 ± 0.06	4.72 ± 0.84*	4.6 ± 0.55*	1.25 ± 0.13^#$^	2.19 ± 0.32^#$&^	10.63 ± 1.52*^#$@&^

*P < 0.05 compared to untreated group, P < 0.05 compared to UCNPs1 group, ^$^P < 0.05 compared to UCNPs2 group, P < 0.05 compared to radiation only group, ^&^P < 0.05 compared to Radiated UCNPs1 group.

In terms of apoptosis, UCNPs1 significantly enhanced Caspase3 expression while reducing BCL2 levels, indicating a stronger pro-apoptotic effect compared to UCNPs2. Following radiation, UCNPs2 maintained relatively high Caspase 3 levels, whereas UCNPs1 exhibited a decline, suggesting differential apoptotic responses between the two formulations. The expression of stemness markers, such as SOX2 and SURVIVIN, was more pronounced in UCNPs1 compared to UCNPs2. However, radiation led to a notable suppression of these markers in both groups, implying that radiation may mitigate their stemness-associated properties. A significant reduction in HIF-α expression was observed in both UCNPs-treated groups, indicating that these formulations suppress hypoxia-related pathways, which could influence cellular adaptation under stress conditions. Additionally, TRPV4, a calcium channel involved in apoptosis and cellular stress, was upregulated in UCNPs1 but remained lower in UCNPs2. After radiation, UCNPs2 demonstrated an increase in TRPV4 expression, while UCNPs1 exhibited a reduction.

Caspase-dependent pathways, known for their precise control of apoptosis, offer clear benefits including effective removal of damaged cells and well-understood mechanisms that serve as targets for cancer therapies^98^. Escaping from programmed cell death or apoptosis is one of the popular theories that explain cancer cell radioresistance. Downregulation of Caspase 3 often indicates a shift from the canonical caspase-dependent apoptotic pathway to alternative mechanisms of cell death or survival^99–101^. Additionally, changes in the balance of BCL2 family proteins might influence the activation of alternative cell death pathways. The activation of these alternative pathways can affect the efficacy of treatments that rely on inducing apoptosis through caspase activation, suggesting the need for strategies that target these alternative mechanisms to overcome potential resistance and improve therapeutic outcomes.

However, their disadvantages include the potential for resistance in cancer cells, which can limit therapeutic effectiveness, and the risk of inflammation and tissue damage due to widespread cell death^102^. Caspase-independent pathways provide alternative mechanisms for cell death that can help overcome resistance to conventional therapies and may result in less inflammatory responses. These pathways, such as autophagy or necroptosis, present opportunities for targeting resistant cells, but they are less well-understood and can lead to variable outcomes or unintended effects. Thus, while caspase-independent pathways offer promising avenues for therapy, they also bring complexities and uncertainties that need careful consideration in therapeutic development. Balancing and understanding both pathways are crucial for designing effective treatments that address diverse cellular responses and therapeutic challenges^103,104^.

Caspase 3 (CASP3) is a key effector in apoptosis, typically activated by cytotoxic agents, radiation, or immunotherapy, and is commonly used to assess cancer therapy effectiveness^104^. However, recent studies have revealed that caspase 3 also plays non-apoptotic roles, contributing to tumor relapse and angiogenesis, suggesting its involvement in tumor progression^105,106^. In a caspase 3 knockout (KO) model of colon cancer, cells displayed reduced clonogenicity, invasiveness, and increased sensitivity to radiation and chemotherapy^107,108^ In vivo, caspase 3 deficient cells showed similar tumor formation rates to untreated and Radiation only (controls) but exhibited heightened sensitivity to radiotherapy and reduced pulmonary metastasis. Mechanistically, caspase 3 knockout cells exhibited altered epithelial-mesenchymal transition (EMT) markers^109,110^, indicating that targeting caspase 3 could enhance therapy sensitivity while reducing invasion and metastasis^104^. The expression of chemotherapeutic resistance markers gens (MDR and ABCC1) was found to be upregulated following treatment with upconversion nanoparticles; therefore, we recommend the inclusion of additional anticancer therapeutic agents that inhibit multi-drug resistance to enhance treatment efficacy.

**Fig. 22:**
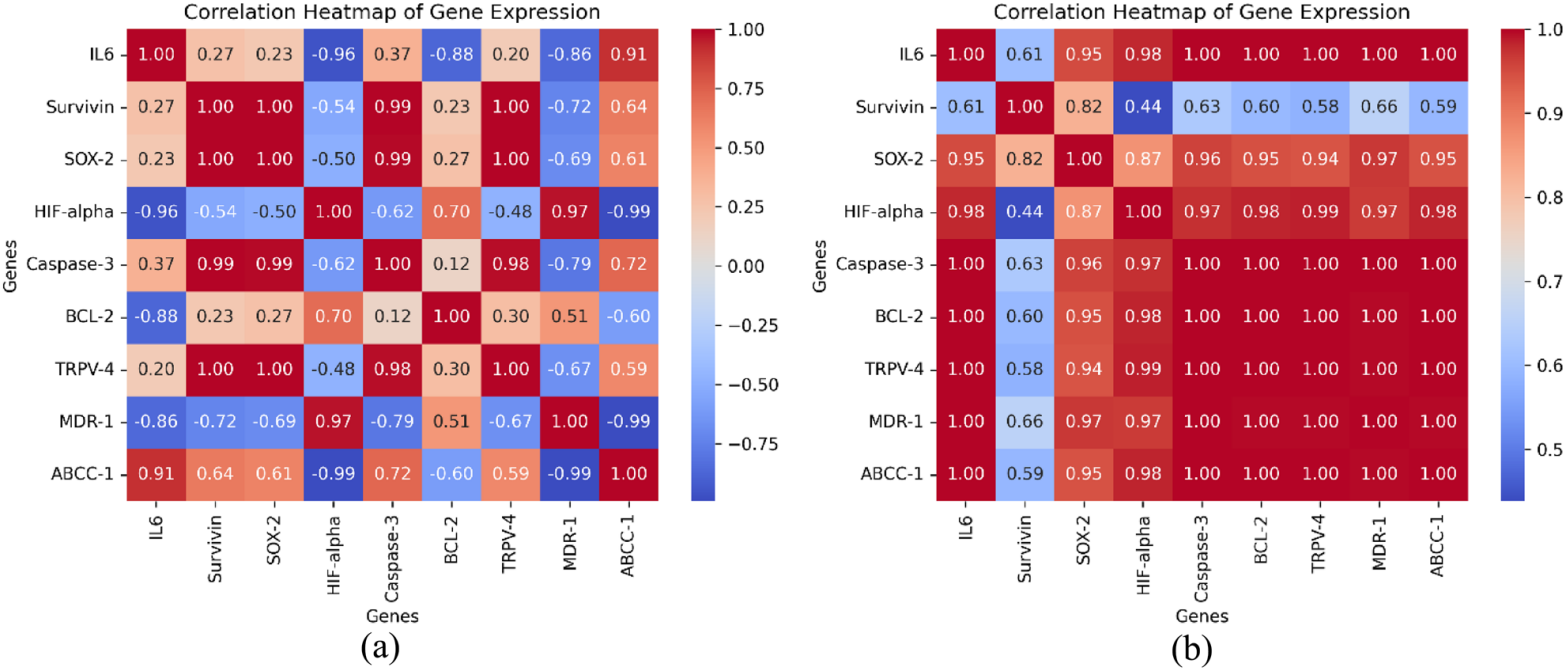
Pearson’s correlation heatmaps showing gene expression patterns across different treatment groups for IL6, Survivin, SOX2, HIF-α, Caspase3, BCL2, TRPV4, MDR1, and ABCC1. (**a**) Correlation matrix following treatment with UCNPs1 and UCNPs2 alone. (**b**) Correlation matrix following treatment with radiated UCNPs1 and radiated UCNPs2. The color gradient reflects the strength and direction of correlation between gene pairs, where red denotes strong positive correlations and blue represents strong negative correlations.

The Pearson’s correlation heatmaps illustrate the relationships among the expression levels of IL6, Survivin, SOX2, HIF-α, Caspase-3, BCL2, TRPV4, MDR1, and ABCC1 under different treatment conditions. UCNPs alone (Fig. 22a) The gene correlations are heterogeneous, with both strong positive and strong negative associations. For example, IL6 shows strong negative correlations with BCL2 (–0.88) and MDR1 (–0.86), but positive correlations with ABCC1 (0.91). Similarly, Caspase-3 correlates negatively with HIF-α (–0.62) but strongly positively with Survivin (0.99). This mixed pattern suggests that UCNPs alone trigger a divergent transcriptional response, where some survival pathways (BCL2, HIF-α, MDR1) are downregulated while pro-inflammatory and apoptotic signals (IL6, Caspase-3) are activated. Radiated UCNPs (Fig. 22b), The correlations become uniformly strong and positive across nearly all gene pairs (r > 0.8). IL6, Caspase-3, HIF-α, and Survivin, for instance, show strong positive clustering, indicating enhanced co-regulation. This suggests that irradiation promotes a more synchronized transcriptional program, likely driving cells towards a consistent stress and apoptosis-related response. The strengthening of correlations between apoptotic markers (Caspase-3) and survival regulators (Survivin, BCL2) indicates cross-talk between pro-death and compensatory survival mechanisms, which is characteristic of stressed cancer cells undergoing treatment pressure. UCNPs alone induce a fragmented and variable transcriptional network, but when combined with irradiation, the cellular response becomes more coordinated and dominated by shared pathways. This transition from heterogeneous to strongly unified correlations implies that irradiation amplifies the biological impact of UCNPs by synchronizing gene regulation, particularly in pathways associated with oxidative stress, apoptosis, and drug resistance.

### Reactive oxygen species (ROS)

**Fig. 23:**
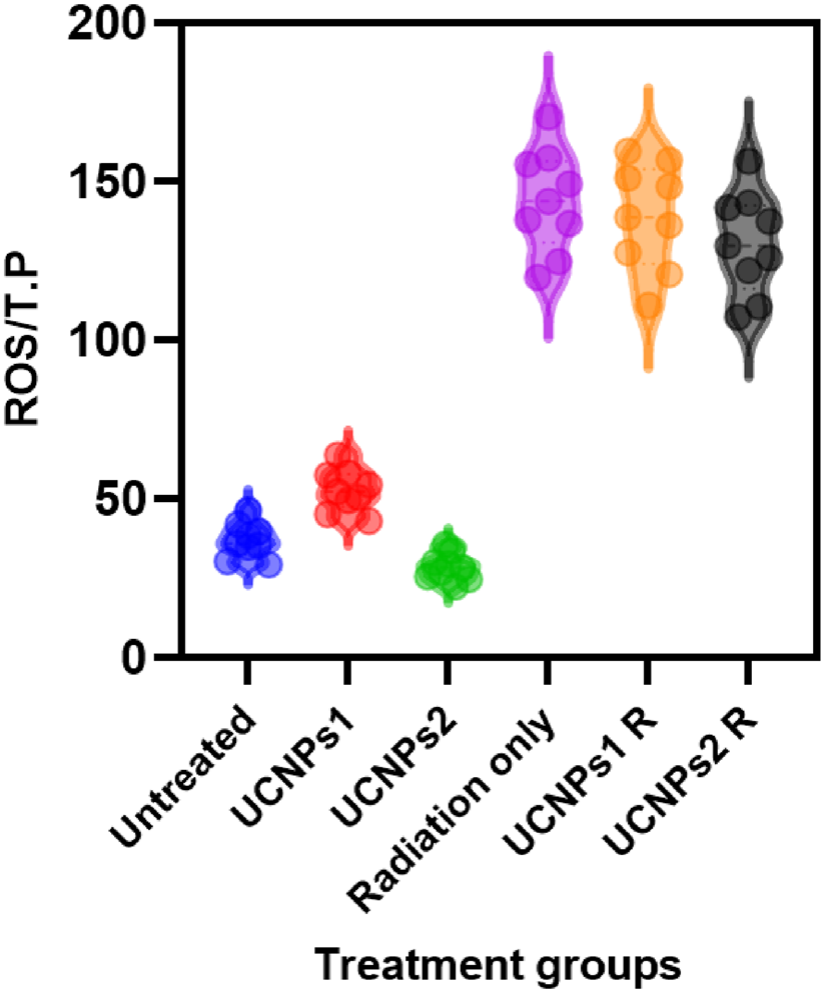
Biochemical analysis of oxidative stress markers in T24 cells across different treatment groups ROS/T.P ratio.

The analysis of reactive oxygen species (ROS) and total protein (T.P) levels reveals distinct effects of UPCNPs and radiation treatments on oxidative stress (Table 7 and Fig. 23). The untreated (control) group exhibits baseline ROS and T.P levels, while UPCNPs1 and UPCNPs2 slightly elevate ROS, indicating increased oxidative stress. Radiation alone (CR) also markedly raises ROS levels, suggesting its role in enhancing oxidative damage^111^. However, when combined with radiation, UPCNPs1 and UPCNPs2 show an elevation in ROS levels, particularly UPCNP1, which results in the highest ROS/T.P ratio.

**Table 7:** Reactive oxygen species and total protein levels.

Sample	Reactive oxygen species (Ros) Pg/ml	Total protein (T.P) g/dl	Ros/T.p
C	229.91 ± 23.38	6.29 ± 0.88	37.017 ± 5.43
UPCNPs1	299.71 ± 24.47	5.74 ± 0.68	52.705 ± 6.52
UPCNPs 2	184.41 ± 17.81	6.53 ± 0.89	28.611 ± 4.26
CR	636.69 ± 43.80	4.46 ± 0.50	143.942 ± 16.15
UPCNPs 1R	584.23 ± 40.83	4.25 ± 0.54	138.89 ± 16.71
UPCNPs 2R	711.24 ± 48.69	5.51 ± 0.70	130.44 ± 15.95

## Conclusions

The study demonstrates that red single-wavelength UCNPs, specifically NaYbF₄:Sm,Nd, exhibit significant potential for cancer therapy by inducing cytotoxicity, modulating gene expression, and generating reactive oxygen species (ROS) in T24 bladder cancer cells. In addition, the suite of physical characterizations performed in this study provides a coherent and complementary picture of the synthesized UCNPs. XRD, TEM, and SAED collectively confirmed the crystalline phase and nanoscale morphology, while XPS and EDX validated the dopant incorporation and oxidation states essential for efficient energy transfer. FTIR, zeta potential, and DLS together demonstrated colloidal stability and surface ligand effects, and photoluminescence measurements directly correlated these structural and compositional features with the observed red emission efficiency. By integrating these findings, the physical characterization results form a consistent structure–composition–property relationship, thereby establishing the physicochemical basis for interpreting the nanoparticles’ biological effects on T24 bladder cancer cells. The nanoparticles’ effects are enhanced under near-infrared (NIR) radiation, leading to increased cytotoxicity and altered gene expression profiles. Lower concentrations of UCNPs are biocompatible, while higher concentrations induce significant cytotoxicity and hemolysis, underscoring the importance of dose optimization. These findings provide valuable insights into the molecular mechanisms of UCNPs and their potential applications in precision oncology, while also emphasizing the need for further research to optimize their therapeutic efficacy, particularly in balancing their cytotoxic effects with their impact on cellular repair mechanisms like wound healing. particularly in inducing apoptosis and modulating gene expression, while also underscoring the need for careful dose optimization to mitigate adverse effects on wound healing and drug resistance. UCNPs1 appears to induce stronger apoptosis, enhance stemness markers, and activate TRPV4 more effectively, while UCNPs2 shows a greater association with inflammation and potential radioresistance. These differences likely stem from their varying compositions, affecting their biological impact on cellular responses before and after radiation exposure. Imaging UCNPs uptake by T24 cancer cells will be addressed in the future studes. Future studies will address incorporating functional apoptosis assays (e.g., Annexin V/PI or TUNEL), complementary migration/proliferation analyses, and cytokine quantification such as ELISA. This study provides a foundation for further research into UCNPs-based therapies in precision oncology.

## Data Availability

The authors declare that the data supporting the findings of this study are available within the paper. Should any raw data files be needed in another format they are available from the corresponding author upon reasonable request.  Additional experimental details, raw data, and processed datasets used in the analysis can be provided to interested researchers.
